# Observation versus coiling for unruptured intracranial aneurysms in octogenarians: A decision-analytic modeling study

**DOI:** 10.1371/journal.pone.0357374

**Published:** 2026-09-01

**Authors:** Hyun Dong Yoo, Jae Guk Kim, Seung Young Chung

**Affiliations:** 1 Department of Neurosurgery, Eulji University Hospital, Eulji University College of Medicine, Daejeon, Republic of Korea; 2 Department of Neurology, Eulji University Hospital, Eulji University College of Medicine, Daejeon, Republic of Korea; Università degli Studi di Napoli Federico II: Universita degli Studi di Napoli Federico II, ITALY

## Abstract

Management of unruptured intracranial aneurysms in patients aged 80 years and older remains uncertain because treatment morbidity, rupture risk, post-rupture outcome, and competing mortality interact. We constructed a competing-risk Markov decision model comparing observation with endovascular coiling in Korean patients at index ages 80, 85, and 90 years; microsurgical clipping and stent-assisted coiling or flow diversion served as additional scenarios. The primary outcome was the undiscounted lifetime cumulative incidence of aneurysm- or treatment-attributable events—treatment morbidity, treatment death, rupture death, or rupture poor outcome—counted as a first-event composite in which all four components carry equal weight regardless of severity, duration, or reversibility. Periprocedural coiling morbidity and mortality were anchored to a U.S. National Inpatient Sample analysis from 2001–2008, and residual post-treatment rupture risk was fixed at 10% of the natural-history rate. Under this original endpoint, median differences favored observation in every age-90 panel and in all male panels at age ≥ 85, and favored coiling in selected age-80 panels and selected age-85 female panels; the probability that coiling was favored ranged from 0.000 to 0.984. A 24-panel, 3%-discounted severity-weighted quality-adjusted analysis was added, together with structural sensitivities for treatment-morbidity recovery, residual rupture risk, and coiling morbidity. Across 30 endpoint-direction comparisons, with each Ma panel mapped to its two legacy marginals, 21 were concordant; all nine discordant comparisons shifted from observation toward coiling, and eight had 95% quality-adjusted uncertainty intervals spanning zero. Median-direction changes under the executed structural sensitivities were confined to modified Rankin Scale-based panels with reference quality-adjusted differences near zero and uncertainty intervals spanning zero. Modeled treatment preference was therefore conditional on endpoint definition and key structural assumptions, including historical treatment-risk estimates and uncalibrated residual post-treatment rupture risk; these model outputs do not establish treatment recommendations for individual patients.

## Introduction

The detection of unruptured intracranial aneurysms (UIA) in elderly patients has increased substantially over the past two decades, driven by broader use of cross-sectional imaging and population aging in countries with advanced imaging access. In Korea, where the population aged over 80 years is projected to continue expanding, clinicians increasingly face the question of whether preventive treatment confers net benefit over observation for an incidentally detected UIA in an octogenarian patient. The decision is difficult because the relevant competing risks — periprocedural treatment complications, residual lifetime rupture risk, and competing mortality from non-aneurysm causes — change rapidly with advancing age, while the published evidence base for outcomes in the ≥ 80 cohort remains limited compared with that for younger UIA cohorts.

Several recent cohort studies have begun to address the octogenarian-specific evidence gap, but the available evidence is heterogeneous in important ways. Brinjikji and colleagues’ analysis of the National Inpatient Sample [1] provided periprocedural complication estimates for elderly clipping and coiling cohorts, but reported short-term in-hospital outcomes rather than lifetime trajectories. Single-center treated aneurysmal subarachnoid hemorrhage (aSAH) cohorts [2] and disposition-based proxy analyses of Medicare claims [3] provide partial information, but use heterogeneous outcome cutoffs (modified Rankin Scale (mRS) > 2, institutional discharge proxy, mRS ≥ 3, mortality only) and grade-mix definitions (all-grade vs poor-grade World Federation of Neurosurgical Societies (WFNS) IV-V). The single-center poor-grade Goldberg cohort [4] provides the closest match to the protocol-primary mRS 4–6 cutoff but is limited to WFNS IV-V patients with predominantly conservative management. The recent Ma cohort [5] reports all-grade ≥ 80 outcomes at longer follow-up but at broader outcome cutoffs. No single published cohort simultaneously satisfies all of the criteria — all-grade ≥ 80, mRS 4–6 cutoff, long-term follow-up, large N — that would constitute the protocol-primary target for a definitive octogenarian aSAH outcome anchor.

Decision-analytic modeling can integrate periprocedural, natural-history, and post-rupture outcome evidence into a unified lifetime framework and quantify the conditions under which preventive treatment may confer net benefit over observation. A strength of this approach is that it makes the dependence of model output on input parameters explicit and allows model-inferred preference to be tested across reasonable parameter perturbations. A corresponding limitation is that the model inherits the heterogeneity of its source evidence; in the octogenarian UIA setting, this heterogeneity is dominated by the post-rupture outcome anchor mismatch described above. Because the available outcome evidence is heterogeneous, such a model must report results across plausible outcome scenarios rather than rely on a single assumption, so that the clinical robustness of any inferred preference can be judged.

We report results in parallel across five heterogeneous post-rupture outcome scenarios. Continuous per-panel differences, uncertainty intervals, and preference probabilities are presented as the primary outputs, with the clinician-interpretable threshold-zone classification retained as a secondary summary. Parameters with weaker evidentiary support—including the SAC/FD proxy and the unanchored vascular access complexity stress test—are reported transparently, and the analysis examines how the endpoint definition and the choice among heterogeneous post-rupture outcome anchors influence the model-inferred preference. Following peer review, we additionally implemented a 24-panel severity-weighted quality-adjusted-life-year analysis, together with structural sensitivity analyses of recovery from treatment morbidity, residual post-treatment rupture risk, and the periprocedural coiling-morbidity anchor, specified in Protocol Amendment v1.5 before any extension results were generated.

## Materials and methods

### Study design and decision question

We constructed a Markov decision-analytic model evaluating observation versus preventive treatment for a clinically detected UIA in Korean patients aged 80 years and older. The perspective was patient-clinical. The original analysis reported an unweighted cumulative-incidence endpoint and excluded utility and cost extensions per protocol. In response to peer review, a severity-weighted extension was implemented under a pre-execution amendment registered before any extension result was generated (Protocol Amendment v1.5), and quality-adjusted life-years are reported alongside the original endpoint rather than in place of it. Cost was not modeled. The model was developed and reported following established recommendations for decision-analytic modeling [6–11].

The primary comparator was observation versus coiling. The main PSA outputs were the continuous per-panel difference in lifetime cumulative incidence between observation and coiling with its uncertainty interval, and the probability that coiling produced the lower incidence; a threshold-zone classification using a ± 0.01 equipoise band is reported as a secondary summary.

Two additional treatment strategies were modeled in parallel: microsurgical clipping and SAC/FD. Clipping was included because it remains a clinical alternative in some elderly UIA cohorts despite its higher morbidity profile in the available evidence base. SAC/FD was modeled as a scenario block representing complex-anatomy aneurysms for which simple coiling is not feasible. Because TES identified no direct ≥ 80 clinical-burden evidence, the SAC/FD scenario was built as a proxy that borrowed the coiling mean with a downweighted effective sample size (ESS), as described below under Parameters and sources and Outcome scenarios. The time horizon was lifetime (terminating at age 100, with a residual five-year computational margin); the base-case discount rate was 0%, with a 3% discounted cumulative-incidence analysis as the key sensitivity.

Three index ages (80, 85, and 90 years) and two sexes (male and female) defined six demographic panels. Crossing these with five post-rupture outcome scenarios (Scenarios A, B, C, E, and F, defined below under Outcome scenarios) yielded 30 base-case panels, each evaluated by probabilistic sensitivity analysis at 10,000 iterations. The setting was Korean clinical practice, with competing mortality drawn from the 2024 Korean Statistical Information Service (KOSIS) life table [12] (see Parameters and sources below); the framework is country-portable by substituting the competing-mortality input.

### Model structure

**Fig 1:**
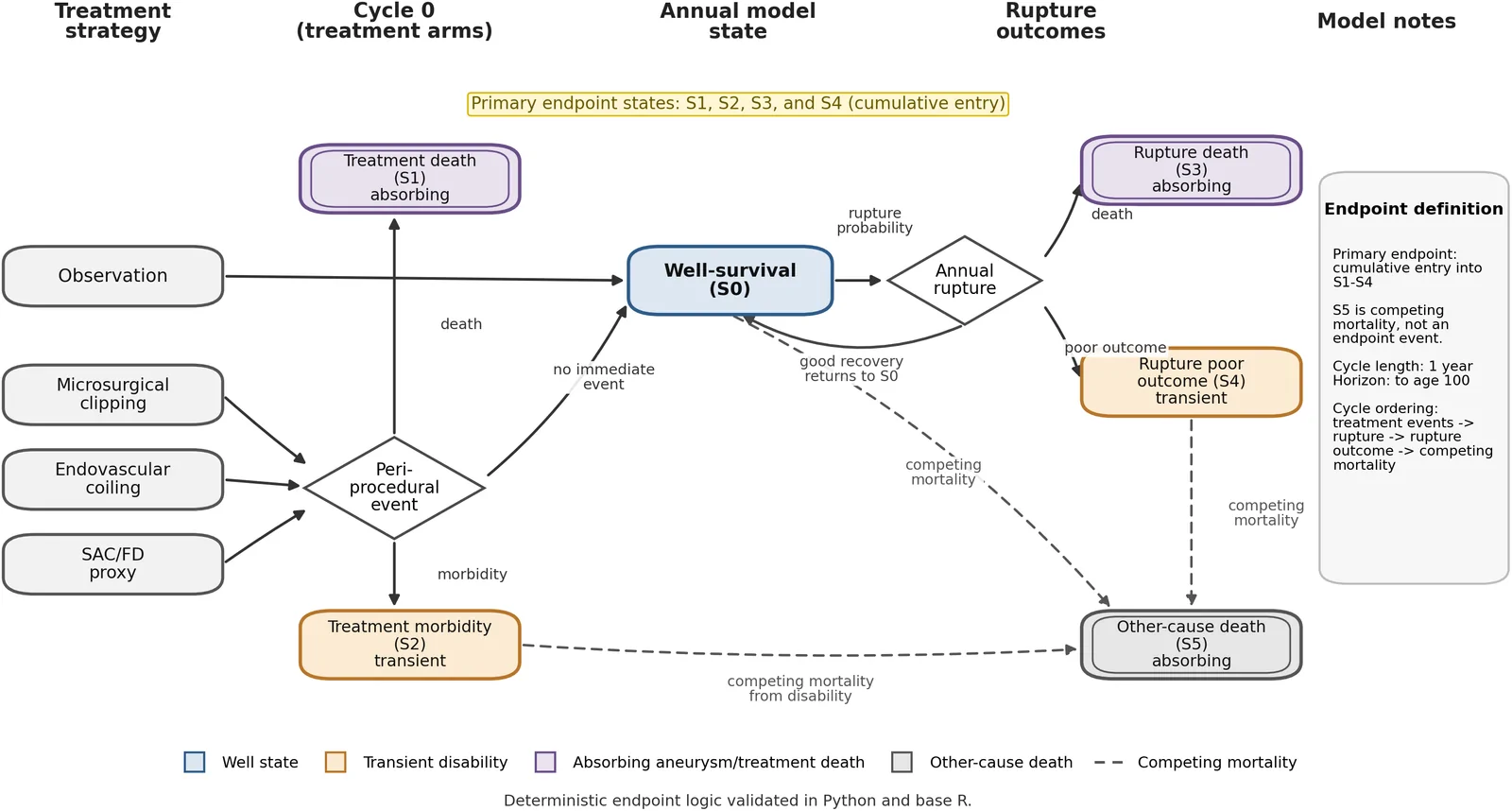
Markov state-transition model schematic. Schematic of the four-strategy Markov decision-analytic model. Each strategy (Observation, Microsurgical Clipping, Endovascular Coiling, and Stent-Assisted Coiling or Flow Diversion as a proxy scenario) uses the same six health states—well-survival, treatment morbidity, treatment death, rupture poor outcome, rupture death, and other-cause death—and the same competing risks, as applicable, including periprocedural events, post-treatment or natural-history rupture, and Korean life-table mortality. Strategy-specific transition probabilities are drawn from the distributions in Table 1. The cycle length is one year, and the lifetime horizon uses the KOSIS terminal age with a residual five-year computational margin. The hierarchical within-cycle event ordering is described under Model structure.

The Markov model consisted of a well-survival state and five mutually exclusive outcome states defined in the prespecified parameter specification. The five outcome states were (1) treatment death, (2) treatment morbidity without death, (3) rupture death, (4) rupture poor outcome without death, and (5) other-cause death. The definition of nonfatal rupture poor outcome was scenario-specific, because the poor-outcome cutoff varied across the five outcome scenarios described below. States 1, 3, and 5 were absorbing. States 2 and 4 represented living-with-disability states that could transition to other-cause death through competing mortality but did not generate additional aneurysm-related events. All cohorts began in state 0 (well-survival). Treatment arms partially exited state 0 in cycle 0 through periprocedural events and could exit progressively in subsequent cycles through residual rupture or competing mortality. Fig 1 shows the model state diagram and the hierarchical cycle ordering.

Each annual cycle followed a prespecified hierarchical event ordering. Step 1: in the treatment arms, cycle 0 represented the treatment cycle, during which the cohort faced treatment mortality and treatment morbidity. Step 2: the observation arm faced the annual natural-history rupture probability in every cycle, anchored to the UCAS-derived elderly cohort of Hishikawa et al. [13]; in the treatment arms, the residual rupture probability was fixed at 10% of that rate. Step 3: rupture events were partitioned by case fatality and by the scenario-specific probability of poor outcome conditional on rupture, with ruptures followed by good recovery returning to well-survival. Step 4: the residual cohort that had not experienced an aneurysm- or treatment-attributable event in that cycle faced the KOSIS competing-mortality probability. Living cohorts in the treatment-morbidity and rupture-poor-outcome states remained subject to competing mortality but were not exposed to further aneurysm risk.

The primary outcome was the undiscounted lifetime cumulative incidence of entry into any of four mutually exclusive aneurysm- or treatment-attributable event states: treatment morbidity, treatment death, rupture death, or rupture poor outcome. This endpoint was an unweighted first-event metric: because a cohort member exited the well-survival state after the first such event and could not re-enter it, each member could experience at most one counted aneurysm- or treatment-attributable event. The cumulative incidence is therefore bounded by 1 and is interpretable as the probability of ever experiencing an aneurysm- or treatment-attributable adverse event over the remaining lifetime. Events were accumulated as they occurred in each annual cycle and retained thereafter, including after any subsequent transition to competing other-cause mortality. This cumulative-incidence definition was adopted before journal submission, replacing an earlier terminal-state occupancy metric that did not retain nonfatal disability events after competing-mortality transitions and therefore approximated cumulative aneurysm/treatment mortality rather than an unweighted first-event cumulative-incidence metric. The principal comparison was observation versus coiling. For each panel and PSA iteration, the cumulative-incidence difference was computed as observation minus coiling. Coiling was classified as favored when this difference exceeded the + 0.01 equipoise band, observation was classified as favored when it was below −0.01, and intermediate differences were classified as equipoise.

### Parameters and sources

All anchored parameters and their primary sources are summarized in Table 1. Parameters were sourced through TES and verified against full text when available, or against abstract or first-page text when full text could not be obtained. Verification status and source-specific caveats are documented in the TES verification record available in the reviewer-accessible OSF materials. Beta distributions were specified by mean and ESS using the conventional method-of-moments approach (Beta α = mean × ESS; Beta β = (1 − mean) × ESS). Lognormal distributions were specified by their log-median and log-scale dispersion; implemented percentile intervals were reported when they differed from nominal bounds. KOSIS competing mortality was treated deterministically in the base case.

**Table 1:** Model parameter specification.

Parameter	Distribution	Central estimate	Dispersion (ESS or 95% interval)	Source (PMID)
**Competing mortality — Korean life table (KOSIS DT_1B41, 2024; treated deterministically, not sampled in PSA)**				
1-year qₓ, age 80–84 (male / female)	Deterministic	0.06548 / 0.03674	—	KOSIS DT_1B41 [12]; five-year age-band qₓ applied as bandwise constant values
85–89 (M / F)	Deterministic	0.12138 / 0.07893	—	KOSIS DT_1B41 [12]
90–94 (M / F)	Deterministic	0.20497 / 0.14969	—	KOSIS DT_1B41 [12]
95–99 (M / F)	Deterministic	0.31479 / 0.25033	—	KOSIS DT_1B41 [12]
100+ (M / F)	Deterministic	1.00000 / 1.00000	—	KOSIS DT_1B41 [12] (terminal qₓ)
**Natural-history rupture risk (annual, age ≥ 80)**				
Annual rupture probability (base)	Lognormal	Median 0.025	Implemented 95% sampling interval 0.018–0.035 ᵉ	Hishikawa 2015 (PMID 26511450) [13]; HR ≥ 80 vs 70–79 = 2.02
Residual rupture after treatment	Fixed fraction	10% of natural-history rate	—	Model assumption
**Periprocedural treatment outcome — Brinjikji 2011 (National Inpatient Sample, ≥ 80; PMID 21441142)**				
Microsurgical clipping — morbidity (poor outcome)	Beta	0.335	ESS 140	Brinjikji 2011 (PMID 21441142) [1]
Microsurgical clipping — mortality	Beta	0.214	ESS 140	Brinjikji 2011 (PMID 21441142) [1]
Endovascular coiling — morbidity	Beta	0.098	ESS 809	Brinjikji 2011 (PMID 21441142) [1]
Endovascular coiling — mortality	Beta	0.024	ESS 809	Brinjikji 2011 (PMID 21441142) [1]
SAC/FD — morbidity (proxy) ᵃ	Beta (scenario-defined)	0.098 (coiling proxy)	ESS 50 base / 100 sensitivity	Coiling proxy; no direct ≥ 80 SAC/FD anchor
SAC/FD — mortality (proxy) ᵃ	Beta (scenario-defined)	0.024 (coiling proxy)	ESS 50 base / 100 sensitivity	Coiling proxy; no direct ≥ 80 SAC/FD anchor
**Post-rupture poor-outcome probability — outcome scenarios (sampled in parallel)**				
Scenario A — treated octogenarian aSAH, mRS > 2	Beta	0.907	ESS 43	Catapano 2021 (PMID 34580755) [2]
Scenario B — Medicare ≥ 80 disposition proxy	Beta	0.640	ESS 1298	Dasenbrock 2018 (PMID 30117766) [3]
Scenario C — poor-grade ≥ 80 mRS 4–6, 6–12 mo ᵇ	Beta	0.900	ESS 20	Goldberg 2018 (PMID 30571422) [4]
Scenario C-acute — Goldberg at discharge ᶜ	Deterministic	1.00 (20/20)	—	Goldberg 2018 (PMID 30571422) [4]; sensitivity only
Scenario E — all-grade ≥ 80 mortality	Beta	0.395	ESS 38	Ma 2024 (PMID 39134300) [5]
Scenario F — all-grade ≥ 80 mRS ≥ 3	Beta	0.474	ESS 38	Ma 2024 (PMID 39134300) [5]
**Vascular access complexity (VAC) — coiling complication multiplier**				
VAC-Low (reference, base case)	Fixed	1.0	—	Reference
VAC-High (stress-test) ᵈ	Lognormal	Median 2.0	Implemented 95% sampling interval ≈1.55–2.58	Unanchored stress-test (Plan B3, scenario-only)
**Model and probabilistic sensitivity analysis (PSA) settings**				
Cycle length	—	1 year	—	—
Time horizon	—	Lifetime (to age 100+)	—	KOSIS terminal age [12]
Discount rate	—	0% base / 3% sensitivity	—	CHEERS / ISPOR [6–11,14]
PSA iterations (random seed)	—	10,000 (seed 2026)	—	Second-order Monte Carlo
Equipoise band	—	±0.01 (cumulative-incidence units)	—	Pre-specified
Primary outcome	—	Undiscounted lifetime cumulative incidence of entry into states 1–4	—	Undiscounted; lifetime horizon (3% discounted cumulative incidence as sensitivity)

Beta priors are specified by the method of moments (α = mean × ESS; β = (1 − mean) × ESS); lognormal distributions are specified by their log-median and log-scale dispersion, and implemented percentile intervals are reported where they differ from the nominal bounds. Abbreviations: aSAH, aneurysmal subarachnoid hemorrhage; CI, confidence interval; ESS, effective sample size; mRS, modified Rankin Scale; PSA, probabilistic sensitivity analysis; qₓ, one-year mortality probability; SAC/FD, stent-assisted coiling or flow diversion; UI, uncertainty interval.

**ᵃ** SAC/FD outcome means are borrowed from the coiling anchor as a proxy; no peer-reviewed ≥ 80 SAC/FD clinical-burden source was identified during targeted evidence synthesis. The effective sample size is scenario-defined (downweighted), not evidence-derived (base ESS 50; sensitivity ESS 100; bounded scenario-range alternative).

**ᵇ** Primary mRS 4–6 anchor; limited to poor-grade (Hunt–Hess / WFNS IV–V) patients, predominantly conservatively managed, single-center, N = 20.

**ᶜ** Goldberg at-discharge value (20/20 = 1.00) is retained only as a deterministic acute-decision sensitivity value; a Beta prior is not used because 20/20 events yield a degenerate distribution.

**ᵈ** VAC-High is an illustrative, unanchored stress-test range (Plan B3), not an evidence-based estimate; the quantitative multiplier could not be anchored to verified literature within project scope. The multiplier was implemented as a lognormal distribution centered at a median of 2.0; nominal bounds of 1.5–2.5 were used to define the log-scale dispersion, and because the distribution was centered at 2.0 rather than at the geometric midpoint of the nominal bounds, the implemented 2.5th–97.5th percentiles were approximately 1.55–2.58.

**ᵉ** The annual rupture-risk distribution was implemented as a lognormal distribution with log-median 0.025. The nominal bounds 0.020–0.040 were used to define the log-scale dispersion; because the distribution was centered at a median of 0.025 rather than at the geometric midpoint of the nominal bounds, the implemented 2.5th–97.5th percentiles were approximately 0.018–0.035.

Three caveats apply to the parameter set in Table 1. First, the SAC/FD morbidity and mortality means were borrowed from the coiling anchor because no direct ≥ 80 SAC/FD clinical-burden evidence was identified during TES verification. These entries are therefore proxy values, and their ESS was downweighted to 50 in the base case and 100 in sensitivity to reflect the absence of direct evidence. Second, the VAC modifier could not be directly anchored to available literature for the ≥ 80 cohort during TES verification. The VAC-High scenario therefore functions as an unanchored stress test rather than a calibrated effect size, and any inference drawn from VAC-High runs must be qualified accordingly. Third, KOSIS qx was treated deterministically in the base case. The prespecified parameter specification documents this convention; the alternative life-table-sampling sensitivity has not been executed in the present analysis.

### Targeted evidence synthesis

Parameter sources were identified through TES rather than a full systematic review. Candidate studies were identified through structured PubMed and Embase searches for cohorts aged 80 years and older. Extracted values were verified against the primary source, using full text when available and abstract or first-page text otherwise; verification status was explicitly downgraded whenever full text could not be obtained. The candidate worklist and verification record are available in the OSF archive and provide an audit trail for each anchor parameter.

Three operational decisions in the TES workflow merit specific disclosure. First, the Goldberg anchor [4] was originally extracted as a Scenario C value of 0.90 mRS 4–6 “at discharge” in an earlier model version but was re-verified during subsequent model development. The correct timepoint is 6–12 month follow-up, as documented in the re-verification addendum in the reviewer-accessible OSF materials, and the final Scenario C anchor reflects this correction. The at-discharge value (20/20 = 1.00) was retained as a deterministic acute-decision sensitivity (Scenario C_acute), not as a Beta prior, because 20/20 events yield a degenerate Beta. Second, the originally planned Dumont 2014 anchor for VAC could not be obtained during TES verification despite multiple search and citation-tracking attempts. A predefined fallback hierarchy was therefore applied, and VAC was demoted from a quantitatively anchored parameter to an unanchored stress-test scenario. Third, the SAC/FD scenario block was constructed as a proxy after TES verification identified no peer-reviewed direct ≥ 80 SAC/FD clinical-burden evidence.

### Post-rupture outcome scenarios

The post-rupture outcome distribution after aSAH in octogenarians is highly heterogeneous across the published evidence base; anchor sources differ in outcome cutoff (mRS 4–6 versus mRS ≥ 3 versus mortality only), case-mix grade (poor-grade only versus all-grade), and timepoint of outcome ascertainment (at discharge versus 6–12 month follow-up). Rather than averaging over these heterogeneous anchors, we treated each as a separate scenario and reported all scenarios in parallel. This makes explicit the dependence of the model-inferred preference on the assumed outcome distribution and preserves structural uncertainty across non-exchangeable outcome definitions.

Five outcome scenarios entered the base case as Beta-distributed PSA priors:

Scenario A (Catapano et al. [2]): treated octogenarian aSAH cohort mRS > 2 = 39/43 = 90.7%, Beta(0.907, ESS = 43). Cutoff is mRS 3–6 (broader than mRS 4–6); treated cohort only.

Scenario B (Dasenbrock et al. [3]): Medicare ≥ 80 aSAH disposition proxy (died/hospice + institutional discharge) = 64%, Beta(0.64, ESS = 1298). Not mRS; disposition-based proxy for post-rupture poor outcome under all-grade ≥ 80 conditions.

Scenario C (Goldberg et al. [4]): single-center ≥ 80–90 aSAH mRS 4–6 at 6–12 month follow-up = 18/20 = 90.0%, Beta(0.90, ESS = 20). This scenario served as the protocol-primary anchor for the mRS 4–6 cutoff. Caveats: WFNS IV-V (poor-grade) only; 80% no aneurysm treatment (i.e., largely conservative management); withdrawal of care 45% in the 80–90 stratum; single-center; small N.

Scenario E (Ma et al. [5]): UPMC ≥ 80 aSAH treated cohort, all-cause mortality at median 17 month follow-up = 15/38 = 39.5%, Beta(0.395, ESS = 38). All-grade cohort (H&H 1–5 mixed). Cutoff is mortality only (mRS 6); not mRS 4–6.

Scenario F (Ma et al. [5]): same cohort, mRS ≥ 3 = 18/38 = 47.4%, Beta(0.474, ESS = 38). Cutoff is mRS 3–6 (broader than mRS 4–6).

Scenario A and Scenario C are intentionally similar in mean (0.907 vs 0.900) but differ in source population: Catapano reflects an all-grade treated cohort with a broader mRS > 2 cutoff, whereas Goldberg reflects a poor-grade WFNS IV-V cohort with the narrower mRS 4–6 cutoff. Their close numerical agreement is therefore reassuring but does not establish that the protocol-primary target (all-grade ≥ 80 mRS 4–6) lies at this value; the cohort-cutoff mismatch is a central limitation that we discuss in the Limitations. Scenario B provides an alternative anchor at the all-grade level via a disposition-based proxy. Scenarios E and F provide the most recent all-grade ≥ 80 evidence at long-term follow-up but at broader outcome cutoffs.

### Probabilistic sensitivity analysis

Probabilistic sensitivity analysis was conducted using second-order Monte Carlo simulation with 10,000 iterations per panel. In each PSA iteration, anchored parameters were drawn from their respective distributions: Beta for proportion parameters, lognormal for rupture risk and the VAC multiplier, and deterministic values for residual rupture and KOSIS competing mortality. The resulting parameter set was then passed through the Markov engine for each strategy. The random seed was fixed at 2026 in the Python reference implementation for reproducibility.

For each panel and PSA iteration, lifetime cumulative incidences were computed for observation, clipping, coiling, and SAC/FD strategies. Three summary statistics were derived: (1) the median observation-minus-coiling difference in cumulative incidence across the 10,000 iterations; (2) the 2.5%−97.5% percentile range of this difference; and (3) the probability that coiling was favored over observation, defined as the proportion of iterations in which the observation-minus-coiling difference exceeded +0.01. Symmetric probabilities for equipoise and for observation-favored were similarly computed.

### Equipoise band and threshold classification

A symmetric equipoise band of ±0.01 (cumulative-incidence units) around zero was used to classify PSA iterations as coiling-favored, equipoise, or observation-favored. The ± 0.01 band corresponds to a one-percentage-point absolute difference in lifetime aneurysm/treatment cumulative incidence and was treated as the smallest difference of clinical relevance for the present decision context. This band is a pragmatic threshold for presentation and was not externally validated; the primary model outputs are the continuous median observation-minus-coiling difference and the continuous probability that coiling was favored, both reported per panel in Table 2. The threshold-zone classification described below is a secondary, clinician-interpretable summary of these continuous outputs rather than a primary result.

**Table 2:** Base-case probabilistic sensitivity analysis under the cumulative-incidence endpoint (30 panels).

Age	Sex	Post-rupture outcome scenario	Median Δ (obs−coil)	95% interval	P(coiling)	P(equipoise)	P(obs)	Zone
80	M	A (Catapano, mRS > 2)	0.0372	−0.0132 to 0.0983	0.851	0.115	0.035	T2
80	M	B (Dasenbrock disposition)	−0.0046	−0.0445 to 0.0438	0.264	0.337	0.399	T4
80	M	C (Goldberg poor-grade mRS 4–6)	0.0364	−0.0154 to 0.0989	0.837	0.122	0.041	T2
80	M	E (Ma all-grade mortality)	−0.0472	−0.0865 to 0.0017	0.012	0.047	0.941	T4
80	M	F (Ma all-grade mRS ≥ 3)	−0.0335	−0.0749 to 0.0179	0.045	0.125	0.830	T4
80	F	A (Catapano, mRS > 2)	0.0733	0.0151 to 0.1443	0.984	0.014	0.002	T1
80	F	B (Dasenbrock disposition)	0.0230	−0.0228 to 0.0790	0.695	0.219	0.086	T2
80	F	C (Goldberg poor-grade mRS 4–6)	0.0724	0.0122 to 0.1443	0.980	0.017	0.003	T1
80	F	E (Ma all-grade mortality)	−0.0293	−0.0745 to 0.0280	0.081	0.156	0.762	T4
80	F	F (Ma all-grade mRS ≥ 3)	−0.0124	−0.0600 to 0.0486	0.220	0.245	0.535	T4
85	M	A (Catapano, mRS > 2)	−0.0062	−0.0478 to 0.0426	0.245	0.324	0.430	T4
85	M	B (Dasenbrock disposition)	−0.0374	−0.0711 to 0.0019	0.009	0.070	0.921	T4
85	M	C (Goldberg poor-grade mRS 4–6)	−0.0068	−0.0491 to 0.0428	0.238	0.321	0.442	T4
85	M	E (Ma all-grade mortality)	−0.0684	−0.1017 to −0.0300	0.000	0.002	0.998	T4
85	M	F (Ma all-grade mRS ≥ 3)	−0.0584	−0.0930 to −0.0181	0.001	0.009	0.990	T4
85	F	A (Catapano, mRS > 2)	0.0203	−0.0267 to 0.0770	0.663	0.231	0.106	T2
85	F	B (Dasenbrock disposition)	−0.0175	−0.0551 to 0.0273	0.105	0.252	0.642	T4
85	F	C (Goldberg poor-grade mRS 4–6)	0.0196	−0.0285 to 0.0775	0.649	0.233	0.118	T2
85	F	E (Ma all-grade mortality)	−0.0558	−0.0924 to −0.0110	0.003	0.020	0.977	T4
85	F	F (Ma all-grade mRS ≥ 3)	−0.0434	−0.0821 to 0.0039	0.014	0.059	0.927	T4
90	M	A (Catapano, mRS > 2)	−0.0396	−0.0738 to −0.0005	0.007	0.060	0.934	T4
90	M	B (Dasenbrock disposition)	−0.0621	−0.0917 to −0.0300	0.000	0.000	1.000	T4
90	M	C (Goldberg poor-grade mRS 4–6)	−0.0399	−0.0753 to −0.0005	0.007	0.057	0.936	T4
90	M	E (Ma all-grade mortality)	−0.0843	−0.1135 to −0.0529	0.000	0.000	1.000	T4
90	M	F (Ma all-grade mRS ≥ 3)	−0.0771	−0.1074 to −0.0444	0.000	0.000	1.000	T4
90	F	A (Catapano, mRS > 2)	−0.0227	−0.0606 to 0.0217	0.069	0.204	0.727	T4
90	F	B (Dasenbrock disposition)	−0.0497	−0.0812 to −0.0139	0.000	0.014	0.986	T4
90	F	C (Goldberg poor-grade mRS 4–6)	−0.0231	−0.0621 to 0.0218	0.067	0.200	0.733	T4
90	F	E (Ma all-grade mortality)	−0.0764	−0.1076 to −0.0416	0.000	0.000	1.000	T4
90	F	F (Ma all-grade mRS ≥ 3)	−0.0678	−0.1001 to −0.0314	0.000	0.001	0.999	T4

Δ = difference in lifetime cumulative incidence of aneurysm/treatment-attributable events (observation minus coiling); positive values favor coiling. Probabilities are from 10,000 PSA iterations. Equipoise band ±0.01. Zones: T1, coiling-dominant (P ≥ 0.95); T2, coiling-mostly-favored (0.50 ≤ P < 0.95); T3, equipoise-dominant; T4, observation-favored (P(obs) > 0.10). P(obs), probability that observation was favored.

Panel-level threshold classification was performed using a prespecified exclusive hierarchical priority rule. Each panel was assigned to exactly one of four zones in the priority order T1 → T4 → T3 → T2. Zone T1 (coiling-dominant) required the probability that coiling was favored over observation to be ≥ 0.95. Zone T4 (observation-favored), assigned with priority over T3 when both rules applied, required a negative median observation-minus-coiling difference and an observation-favored probability of more than 0.10. Zone T3 (equipoise-dominant) required the equipoise probability to exceed 0.50 with a non-negative median difference. Zone T2 (coiling-mostly-favored) captured the remaining panels with 0.50 ≤ probability of coiling-favored <0.95. This priority order ensures that panels with observation-favored medians are not misclassified as equipoise even when the equipoise probability is also high.

### Post hoc robustness analyses

Because two clinically important uncertainties were not fully resolved by the base-case parameterization—aneurysm rupture-risk heterogeneity and the possibility of lower contemporary coiling morbidity—we performed additional post hoc robustness analyses (S1 Table). First, we repeated the observation-versus-coiling comparison under fixed annual rupture-risk scenarios of 0.5%, 1.0%, 2.5%, and 5.0%, while preserving the remaining probabilistic parameter structure. Second, we performed a coiling-morbidity threshold analysis in which the nonfatal coiling morbidity probability was reduced from the base-case value (9.8%) to hypothetical values of 5% and 3% (coiling mortality and other inputs unchanged), to probe the possibility that contemporary coiling morbidity may be lower than the historical periprocedural anchor. These analyses used the same competing-risk engine, equipoise band, and threshold-zone classification as the base case; the severity-weighted analysis added in revision reports continuous differences and uncertainty intervals without a threshold-zone classification. The original-endpoint robustness analyses were intended to assess the qualitative sensitivity of that endpoint to clinically plausible structural uncertainty and were not used to redefine the base-case model.

### Severity-weighted quality-adjusted life-year analysis

The severity-weighted extension comprised 24 panels: 18 modified Rankin Scale (mRS)-based panels from the Ma, Catapano, and Goldberg sources, each crossed with the same three index ages and two sexes, and six secondary disposition-based panels from Dasenbrock. The four sources were analyzed separately and were not pooled. For the mRS-based sources, mutually exclusive post-rupture outcome categories were sampled jointly from a Jeffreys Dirichlet posterior with alpha equal to the observed count plus 0.5, using the counts reported by each source (15/3/20 for Ma, 14/25/4 for Catapano, and 18/0/2 for Goldberg); category labels were retained as each source defined them rather than harmonized across clinically different outcome definitions.

Utilities combined Korean attained-age EQ-5D values reported by Hwang et al. [15] with utility-weighted mRS values derived using a Korean EQ-5D-5L tariff, reported alongside other national tariffs by Irie et al. [16]. Attained-age well-state utilities were 0.845 at ages 80–84, 0.792 at ages 85–89, and 0.741 at age 90 or above, applied as a step function to both sexes. The Korean-tariff utilities for mRS 0–6 were 0.90, 0.85, 0.76, 0.65, 0.41, 0.24, and 0. The primary mapping applied the relative mRS decrement to the attained-age baseline as u(a,c) = u_Hwang(a) × u_Irie,Korea(c) / 0.90; where a source reported an mRS interval rather than a single score, utilities were averaged equally across the scores in that interval, giving 0.837 for mRS 0–2, 0.433 for mRS 3–5, 0.325 for mRS 4–5, and 0.650 for mRS 3. The primary mapping therefore combined an EQ-5D-3L attained-age baseline with relative mRS weights derived using an EQ-5D-5L Korean tariff; this cross-version normalization was treated as a structural assumption. Utility values were fixed point estimates and were not sampled in the probabilistic analysis. Disposition states were mapped to proxy utilities of 0.837 for home, 0.530 for institutional care, 0.325 for inpatient care, and 0.240 for hospice, anchored at the day of rupture with all patients in inpatient care and interpolated linearly to the source-reported 60-, 365-, and 730-day observations; these mappings are structural proxies rather than validated equivalences.

Treatment morbidity was modeled by duration and persistence rather than assuming all nonfatal treatment morbidity to be permanent. The reference configuration treated 50% of nonfatal treatment morbidity as permanent and the remainder as temporary for 6 months, an operational structural choice rather than an empirically estimated recovery distribution; both used the institutional-care proxy utility before attained-age adjustment, and temporary morbidity recovered to the post-treatment well state if the patient remained alive and rupture-free. Quality-adjusted life-years were discounted at 3% annually, and each panel was evaluated over 10,000 paired probabilistic iterations in which the observation and coiling arms drew on the same underlying parameter sample. The primary output was the difference in discounted quality-adjusted life-years, coiling minus observation, so that positive values favor coiling; medians, 95% uncertainty intervals, and the probability that the difference exceeded zero are reported. Three amendment-specified sensitivity analyses varied recovery from treatment morbidity, residual post-treatment rupture risk, and the nonfatal coiling-morbidity anchor, in the mRS-based panels only and without factorial crossing. Coiling-morbidity levels were evaluated as fixed one-way structural settings rather than resampled around each level, whereas coiling mortality retained its legacy probabilistic specification. Before the first rupture, natural-history rupture risk remained active in the observation arm and residual rupture risk in surviving treated states. After the first rupture, no further aneurysm-related events were modeled; the mRS-based post-rupture states were thereafter subject only to background mortality, while the disposition family followed its source-defined occupancy trajectory through day 730 before background mortality resumed.

### Software and code availability

The cumulative-incidence model was implemented in Python as the reference probabilistic engine and independently checked in base R under deterministic mean-parameter inputs. The Python implementation loaded the verified KOSIS mortality inputs, sampled the parameter distributions specified in Table 1, ran 10,000 probabilistic sensitivity-analysis iterations per panel, and wrote the panel-level summaries. The base-R implementation reproduced the same cumulative-incidence transition logic but was not used as a second probabilistic sampling engine. For computational efficiency, both implementations combined treatment death and rupture death into a single absorbing aneurysm/treatment-attributable death index; this is algebraically equivalent to the two conceptual states shown in Fig 1 and does not affect the cumulative-incidence calculation. The Python and R implementations, JSON and CSV outputs, and figure-generation scripts are openly available in the cited OSF repository. The severity-weighted extension was implemented as a separate layer over the unmodified frozen engine and is available in the same repository, together with the evaluation registry, the draw manifest, and the per-evaluation result files. Four reference evaluations were completed in a second Python/NumPy environment using the same frozen code and draw manifest; before the outputs were combined, cross-environment controls in both the mRS and disposition outcome families reproduced the complete result objects exactly.

### Registration and protocol amendments

The project had an initial OSF protocol record and was first registered in PROSPERO on 10 May 2026 (CRD420261391594; https://www.crd.york.ac.uk/PROSPERO/view/CRD420261391594) as a systematic-review and threshold-based decision-analysis pathway. During pre-submission feasibility assessment, after pilot work but before completion of formal systematic-review searching, screening, risk-of-bias assessment, certainty assessment, or meta-analysis, the systematic-review/meta-analysis pathway was discontinued and the project was amended to a decision-analytic Markov modeling study using targeted evidence synthesis to parameterize a finite set of model inputs. PROSPERO version 2.0, published on 15 June 2026, records this methodological amendment and the discontinued status of the registered review pathway. Accordingly, the final manuscript is not a completed systematic review or meta-analysis: no random-effects meta-analysis, publication-bias assessment, formal risk-of-bias or certainty assessment, or dual independent PRISMA-style screening was performed. The severity-weighted extension was specified in Protocol Amendment v1.5, registered and frozen before any extension result was generated. During revision, execution of the registered utility-extension plan was restricted to the four analysis layers that directly addressed the reviewer’s principal concerns: the 24-panel reference QALY analysis, recovery-trajectory sensitivity (72 evaluations), residual post-treatment rupture-risk sensitivity (54 evaluations), and coiling-morbidity sensitivity (36 evaluations), for 186 evaluations in total. The registered discount-rate (48 evaluations), alternative utility-form (36 evaluations), within-bin utility (12 evaluations), and institutional-utility (6 evaluations) sensitivity layers were not executed; their omission, totaling 102 evaluations, is reported as a deviation from the registered analysis plan. No results from these unexecuted layers were generated or used in the interpretation.

The Stage 1 OSF registration (https://doi.org/10.17605/OSF.IO/BXDSG) documented the initial research plan. Protocol Amendment v1.3 (dated 2026 May 17) documented the methodological pivot to a decision-analytic study with TES parameterization, and Protocol Amendment v1.4 documented the pre-submission revision of the primary endpoint from terminal-state occupancy to undiscounted lifetime cumulative incidence. Reviewer-accessible OSF materials include the parameter table, analysis code, cumulative PSA output summaries, figure source files, the TES candidate worklist and verification record, and protocol amendments, and are openly available on the Open Science Framework at https://osf.io/vnzrt/.

### Artificial intelligence tools and technologies

During manuscript preparation and revision, the authors used Claude (Anthropic) and ChatGPT (OpenAI), generative artificial-intelligence large language model assistants, to support language editing, formatting, reference and consistency checking, code-review and implementation support, verification of numerical outputs and tables, document quality control, and the preparation of submission-related text. AI-generated outputs were reviewed and checked by the authors against the underlying code, analysis outputs, source materials, and manuscript text as applicable. The tools were not used as autonomous sources of scientific claims, to generate or alter primary data, or to make independent clinical or statistical interpretations. All model code, parameter values, numerical results, tables, figures, citations, and manuscript statements were reviewed, verified, and approved by the authors, who take full responsibility for the accuracy, integrity, and originality of all content in the manuscript and its supporting files.

### Ethics statement

This study used only published aggregate data and did not involve individual-level patient data; therefore, institutional review board approval and informed consent were not applicable.

## Results

### Model implementation

The Markov model and probabilistic sensitivity analysis were implemented in Python as the reference probabilistic engine. The cumulative-incidence endpoint was independently validated in base R under deterministic mean-parameter inputs. This deterministic cross-check reproduced the Python cumulative-incidence outputs to machine precision: maximum absolute differences were 4.71 × 10^−^¹³ for observation, 4.57 × 10^−^¹³ for coiling, and 4.94 × 10^−^¹³ for the observation-minus-coiling difference. Because the probabilistic sampling streams were not identical across languages, PSA results were reported from the Python reference implementation.

### Base-case PSA results

Across the 30 base-case panels, the probability that coiling was favored over observation ranged from 0.000 to 0.984. The median difference in lifetime cumulative incidence (observation minus coiling) was positive—favoring coiling—in selected age-80 panels and in age-85 females under Scenarios A and C, and was negative—favoring observation—in the remaining age-80 panels, in all panels at index age 90, and in all male panels at age ≥ 85. For example, at index age 80 in females under Scenario A (Catapano), the probability that coiling was favored was 0.984 (median difference +0.073); at index age 90 in males under Scenario E (Ma all-cause mortality), it was 0.000 (median difference −0.084). Panel-level estimates with 95% intervals are given in Table 2.

### Threshold-zone classification

As a secondary summary, the prespecified classification assigned 2 panels to T1, 5 to T2, none to T3, and 23 to T4 (Table 3). Zone assignments are conditional on the ±0.01 equipoise band and hierarchical classification rule; the continuous estimates and uncertainty intervals in Table 2 remain the primary results. The panels assigned to zones T1–T2 under the original endpoint are listed in S2 Table. That listing restates the continuous estimates in Table 2 under one specific ±0.01 band and threshold set; it is not an independent finding.

**Table 3:** Secondary threshold-zone summary under the cumulative-incidence endpoint. Derived from the continuous per-panel estimates in Table 2 under a ± 0.01 equipoise band; provided for interpretability, not as a primary result.

Threshold zone	n (%)	Panel membership
T1 — coiling-dominant (P ≥ 0.95)	2 (7%)	80F-A, 80F-C
T2 — coiling-mostly-favored (0.50 ≤ P < 0.95)	5 (17%)	80M-A, 80M-C, 80F-B, 85F-A, 85F-C
T3 — equipoise-dominant	0 (0%)	—
T4 — observation-favored (P(obs) > 0.10)	23 (77%)	80M-B, 80M-E, 80M-F, 80F-E, 80F-F, 85M-A, 85M-B, 85M-C, 85M-E, 85M-F, 85F-B, 85F-E, 85F-F, 90M-A, 90M-B, 90M-C, 90M-E, 90M-F, 90F-A, 90F-B, 90F-C, 90F-E, 90F-F

### Age and outcome-scenario dependence

**Fig 2:**
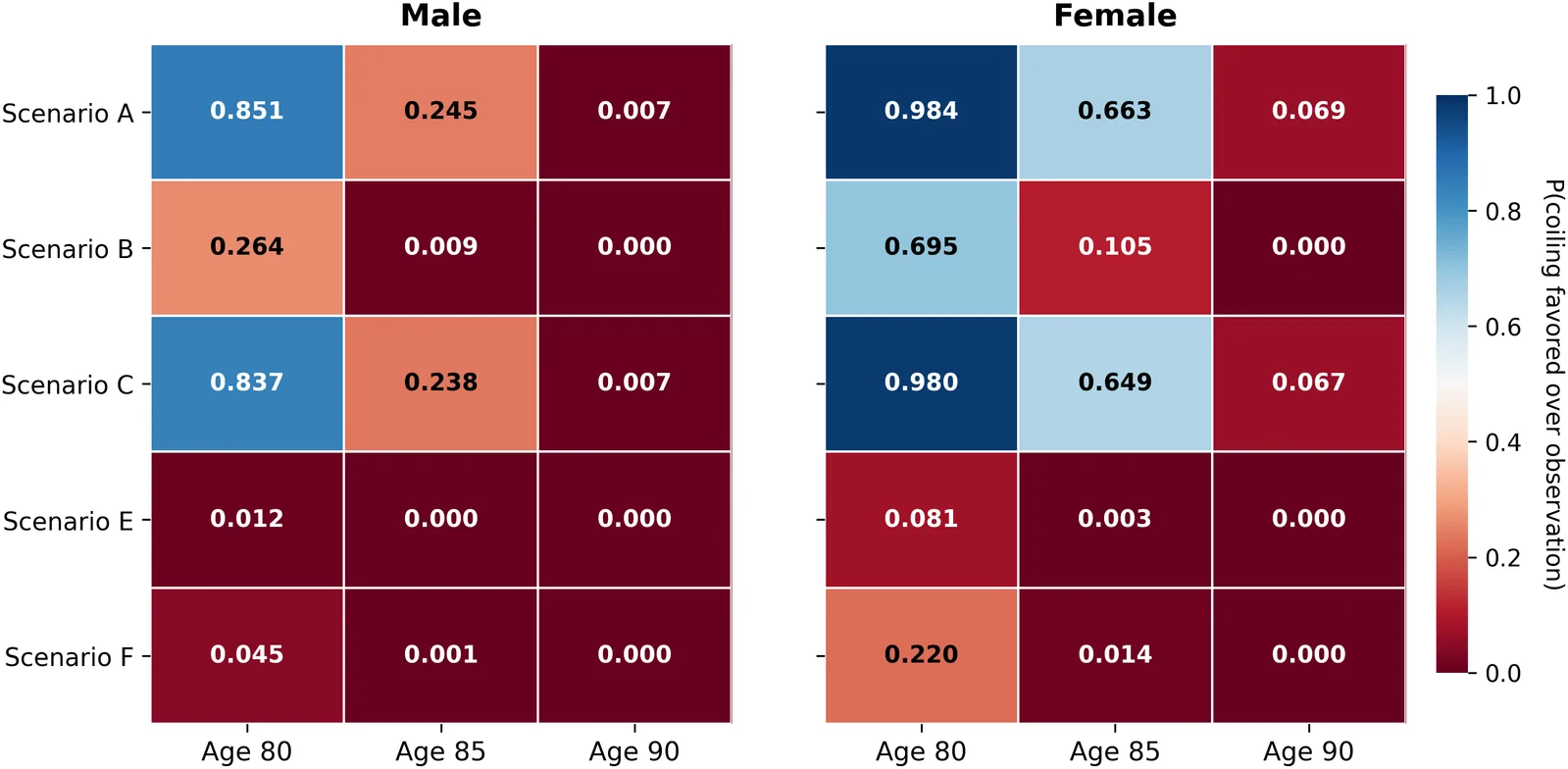
Probability that coiling was favored over observation, by age and post-rupture outcome scenario, faceted by sex. Heatmap of the probability that coiling was favored over observation — defined as an observation-minus-coiling difference in the lifetime cumulative incidence of aneurysm- or treatment-attributable events exceeding the + 0.01 equipoise band — across the 30 base-case panels, shown in two panels by sex (male, left; female, right). Within each panel, rows are the five post-rupture outcome scenarios (Scenario A, Catapano; B, Dasenbrock; C, Goldberg; E, Ma all-cause mortality; F, Ma mRS ≥ 3) and columns are the three index ages (80, 85, 90 years). Probabilities are estimated from probabilistic sensitivity analysis at 10,000 iterations per panel and are overlaid numerically in each cell. Cell color follows a continuous diverging scale: blue indicates coiling favored, white indicates equipoise (probability ≈ 0.5), and red indicates observation favored. Coiling-favored cells are confined to index age 80 and to age-85 females under the high post-rupture poor-outcome scenarios.

**Fig 3:**
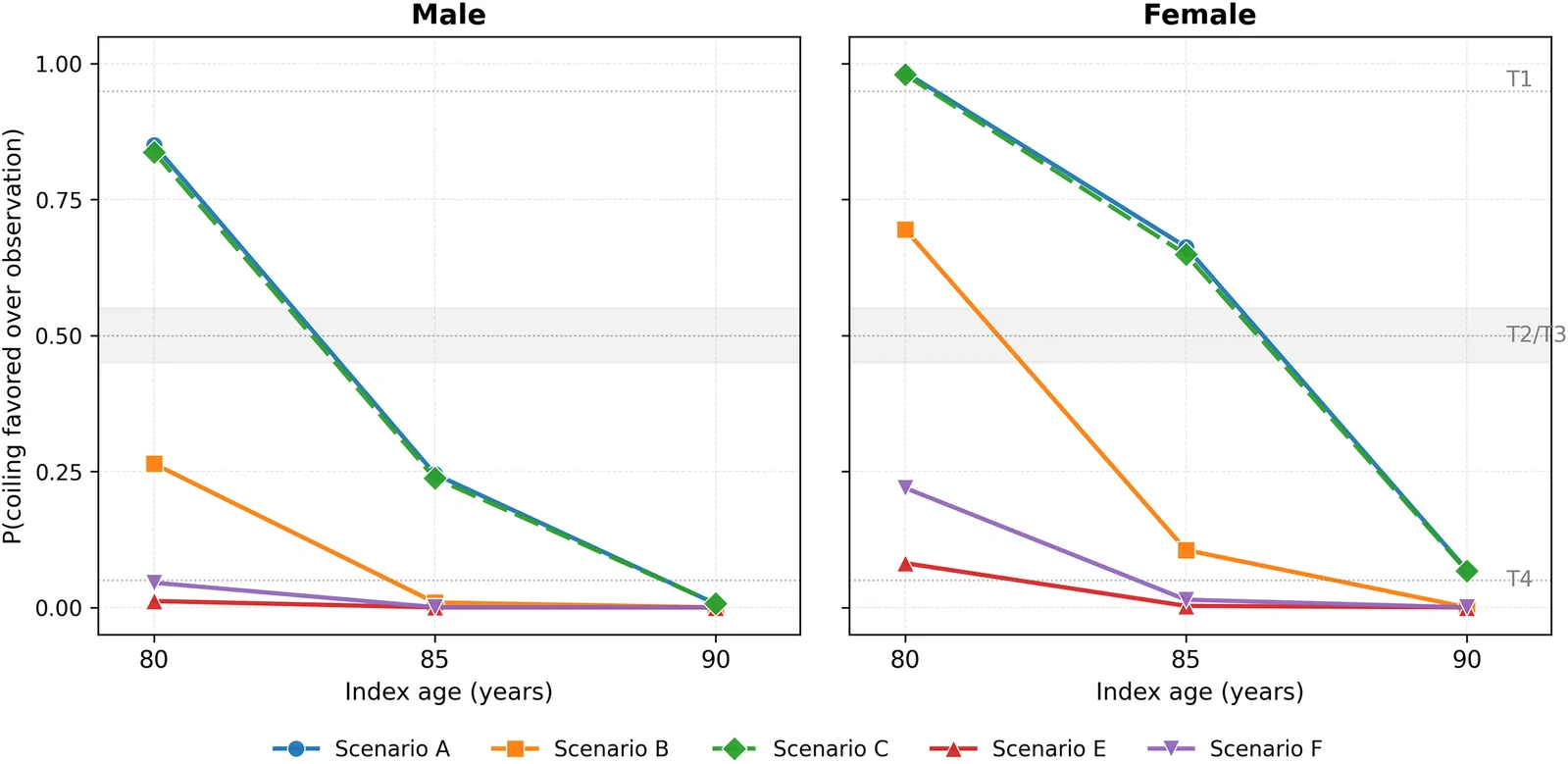
Probability that coiling was favored over observation as a function of index age, stratified by post-rupture outcome scenario. Each panel plots the probability that coiling was favored over observation (an observation-minus-coiling difference in the lifetime cumulative incidence of aneurysm- or treatment-attributable events exceeding the ± 0.01 equipoise band) against index age (80, 85, 90 years), with one curve per post-rupture outcome scenario (Scenario A, Catapano; B, Dasenbrock; C, Goldberg; E, Ma all-cause mortality; F, Ma mRS ≥ 3). Panels are faceted by sex (male, left; female, right). Probabilities are estimated from probabilistic sensitivity analysis at 10,000 iterations per panel. Horizontal reference lines indicate the prespecified threshold-zone boundaries described in the Methods and are shown only as secondary interpretive aids; the continuous panel-level probabilities remain the primary quantities displayed in the figure. Across all five scenarios, the probability that coiling was favored over observation declined with increasing index age; the probability was highest and declined most steeply under the high post-rupture poor-outcome scenarios (A and C), and was uniformly low across all index ages under the Ma-anchored all-grade scenarios (E and F).

The base-case heatmap (Fig 2) showed that the probability that coiling was favored was highest under the high post-rupture poor-outcome scenarios (Scenario A, Catapano; Scenario C, Goldberg) and lowest under the Ma-anchored all-grade scenarios (Scenario E, all-cause mortality; Scenario F, mRS ≥ 3), with coiling-favored cells confined to index age 80 and to age-85 females. The probability that coiling was favored declined steeply with increasing index age (Fig 3): at index age 80 it remained above 0.50 under the higher post-rupture poor-outcome scenarios (Catapano, Dasenbrock, and Goldberg) in at least one sex; by index age 85 it exceeded 0.50 only in females under Scenarios A and C; and at index age 90 it was below 0.10 in every scenario and both sexes.

### Strategy-level comparison

Within the treatment arms, the rank order from lowest to highest lifetime cumulative incidence was generally coiling < SAC/FD < clipping, reflecting the Brinjikji [1] ≥ 80 anchor estimates of treatment morbidity (clipping 33.5% vs coiling 9.8%) and treatment mortality (clipping 21.4% vs coiling 2.4%). The SAC/FD scenario tracked the coiling estimate closely by construction, because the SAC/FD outcome distribution borrowed the coiling mean as a proxy in the absence of a direct ≥ 80 SAC/FD clinical-burden anchor (see Methods). Its 95% interval was wider because the SAC/FD ESS was set to 50 (base case) rather than the coiling ESS of 809.

### Sensitivity analysis

In the 3% discounted cumulative-incidence sensitivity analysis, the probability that coiling was favored was lower than in the undiscounted base case in every coiling-favored panel. Examples included index-age-80 males under Scenario A (0.851 to 0.610), index-age-80 females under Scenario A (0.984 to 0.896), and index-age-85 females under Scenario A (0.663 to 0.400). No panel moved from observation-favored to coiling-favored under discounting. This pattern was expected: discounting down-weights observation’s predominantly future, rupture-related events relative to coiling’s immediate periprocedural morbidity, so the limited support for coiling weakened rather than strengthened. Two further sensitivity analyses are reported under the cumulative-incidence endpoint in the Supplementary Material. In the VAC-High stress test of the treatment arms, no panel was coiling-favored, and the probability that coiling was favored fell to ≤ 0.13 in every panel. The SAC/FD effective-sample-size analysis had only a minor effect on the secondary observation-versus-SAC/FD comparison (maximum change in probability 0.08).

Two post hoc robustness analyses are summarized here, with full panel-level detail in S1 Table. These analyses showed that the model-inferred preference was highly sensitive to the assumed annual rupture risk and to the assumed lower coiling morbidity. Under fixed annual rupture-risk scenarios, no panel favored coiling at 0.5% or 1.0% (0 of 30); the coiling-favored set was unchanged at 2.5% (7 of 30, the same panels as the base case, consistent with the base-case lognormal rupture-risk median of 2.5%); and coiling was favored in 24 of 30 panels at 5.0%. In the coiling-morbidity threshold analysis, reducing the nonfatal coiling morbidity probability from the base-case 9.8% to hypothetical values of 5% and 3% (coiling mortality unchanged) increased the number of coiling-favored panels to 20 of 30 and 24 of 30, respectively. Thus, observation was favored in most panels under base-case assumptions, whereas coiling became favored in most panels once a higher annual rupture risk or a lower coiling morbidity was assumed; coiling-favored conditions expanded monotonically with higher assumed rupture risk or lower assumed coiling morbidity. Two specific aspects were nonetheless stable. First, observation remained favored in every panel at the lowest plausible annual rupture risks (0.5% and 1.0%). Second, a consistent subset of six panels — predominantly the index-age-90 panels and the all-grade post-rupture outcome scenarios (Scenarios E and F) — remained observation-favored or in equipoise even under the most coiling-favorable assumptions tested (5.0% annual rupture risk or 3% coiling morbidity).

## Severity-weighted (QALY) analysis

### Reference analysis

Under the reference configuration, the severity-weighted difference in discounted lifetime QALYs (coiling minus observation) ranged from −0.068 to +0.441 across the 24 panels. The 95% uncertainty interval excluded zero in 8 of the 18 mRS-based panels and in 3 of the 6 disposition-based panels. Coiling was favored with an interval excluding zero in both age-80 panels under the Catapano treated scenario (+0.165, 95% UI + 0.023 to +0.339; + 0.285, + 0.089 to +0.526) and in four Goldberg panels (+0.274, + 0.107 to +0.493 and +0.441, + 0.210 to +0.741 at age 80; + 0.102, + 0.006 to +0.218 and +0.186, + 0.055 to +0.352 at age 85). Observation was favored with an interval excluding zero in the two age-90 Ma panels (−0.068, −0.110 to −0.024; −0.064, −0.118 to −0.003) and in the two age-90 disposition panels (−0.066, −0.101 to −0.035; −0.052, −0.099 to −0.005). Coiling was also favored with an interval excluding zero in the age-80 female disposition panel (+0.164, 95% uncertainty interval +0.006 to +0.353). In the remaining 13 panels the interval spanned zero. Panel-level estimates are shown in Table 4.

**Table 4:** Discounted quality-adjusted life-years by panel under the reference configuration. Values are medians with 95% uncertainty intervals from 10,000 probabilistic iterations, discounted at 3% annually. ΔQALY is coiling minus observation, so a positive value favors coiling. P is the probability that ΔQALY exceeds zero. Panels are grouped by outcome-definition source following the reporting hierarchy of Protocol Amendment v1.5; the four sources are not interchangeable estimates of one quantity and are not pooled.

Panel	Observation QALY	Coiling QALY	ΔQALY (95% UI)	P(ΔQALY > 0)
**Ma — primary mRS outcome reference**				
80, male	5.531 (5.366 to 5.646)	5.525 (5.454 to 5.578)	−0.006 (−0.129 to +0.148)	0.459
80, female	6.728 (6.502 to 6.889)	6.783 (6.693 to 6.849)	+0.055 (−0.115 to +0.267)	0.731
85, male	3.799 (3.714 to 3.861)	3.744 (3.696 to 3.780)	−0.055 (−0.128 to +0.029)	0.093
85, female	4.685 (4.563 to 4.773)	4.654 (4.594 to 4.699)	−0.031 (−0.130 to +0.086)	0.290
90, male	2.517 (2.475 to 2.547)	2.449 (2.417 to 2.472)	−0.068 (−0.110 to −0.024)*	0.002
90, female	3.087 (3.028 to 3.130)	3.023 (2.984 to 3.052)	−0.064 (−0.118 to −0.003)*	0.021
**Catapano — treated, mixed severity**				
80, male	5.340 (5.149 to 5.478)	5.505 (5.431 to 5.559)	+0.165 (+0.023 to +0.339)*	0.990
80, female	6.470 (6.208 to 6.665)	6.754 (6.664 to 6.822)	+0.285 (+0.089 to +0.526)*	0.999
85, male	3.690 (3.589 to 3.765)	3.733 (3.683 to 3.769)	+0.041 (−0.039 to +0.138)	0.834
85, female	4.537 (4.391 to 4.644)	4.638 (4.577 to 4.683)	+0.101 (−0.009 to +0.238)	0.961
90, male	2.458 (2.408 to 2.495)	2.442 (2.411 to 2.466)	−0.015 (−0.061 to +0.034)	0.259
90, female	3.010 (2.940 to 3.061)	3.015 (2.976 to 3.044)	+0.005 (−0.055 to +0.074)	0.555
**Goldberg — poor-grade**				
80, male	5.216 (4.974 to 5.388)	5.491 (5.418 to 5.549)	+0.274 (+0.107 to +0.493)*	1.000
80, female	6.293 (5.965 to 6.536)	6.735 (6.643 to 6.808)	+0.441 (+0.210 to +0.741)*	1.000
85, male	3.624 (3.496 to 3.717)	3.726 (3.677 to 3.762)	+0.102 (+0.006 to +0.218)*	0.981
85, female	4.442 (4.259 to 4.574)	4.627 (4.567 to 4.675)	+0.186 (+0.055 to +0.352)*	0.998
90, male	2.423 (2.360 to 2.470)	2.439 (2.407 to 2.463)	+0.016 (−0.036 to +0.077)	0.717
90, female	2.961 (2.870 to 3.026)	3.010 (2.971 to 3.040)	+0.049 (−0.020 to +0.134)	0.905
**Dasenbrock — disposition-based, secondary outcome family**				
80, male	5.459 (5.327 to 5.559)	5.518 (5.446 to 5.571)	+0.058 (−0.054 to +0.189)	0.836
80, female	6.606 (6.410 to 6.755)	6.770 (6.681 to 6.836)	+0.164 (+0.006 to +0.353)*	0.980
85, male	3.775 (3.712 to 3.821)	3.742 (3.694 to 3.777)	−0.033 (−0.096 to +0.034)	0.159
85, female	4.634 (4.535 to 4.708)	4.649 (4.589 to 4.693)	+0.015 (−0.074 to +0.115)	0.623
90, male	2.515 (2.491 to 2.532)	2.449 (2.417 to 2.472)	−0.066 (−0.101 to −0.035)*	0.000
90, female	3.074 (3.033 to 3.104)	3.022 (2.983 to 3.051)	−0.052 (−0.099 to −0.005)*	0.015

* 95% uncertainty interval excludes zero.

QALY, quality-adjusted life-year; UI, uncertainty interval. Probabilities are rounded to three decimals; a displayed value of 1.000 denotes a probability above 0.9995, not certainty.

The Dasenbrock block is a secondary disposition-based outcome family. Its outcome states are discharge dispositions mapped to utilities through a reasoned proxy crosswalk, not a validated equivalence, and its rupture components are not separately identifiable.

No aggregate count across the 24 panels is reported. The panels are not co-equal replicates: the four sources differ in population, severity mix and outcome definition, and the reporting hierarchy of Protocol Amendment v1.5 designates Ma as the primary mRS reference. A tally of the form “coiling favored in X of 24” would imply an exchangeability that the design explicitly rejects.

Discounting differs between endpoints. This table discounts at 3% annually. The original cumulative-incidence analysis is undiscounted in its base case, with a 3% analysis as its key sensitivity; values from the two endpoints are not directly comparable.

### Component-specific outcomes

Reporting the four adverse first-event components separately clarifies why endpoint weighting can change interpretation. In the Ma 80F panel, which was discordant between the original event endpoint and the reference QALY endpoint, the coiling arm had median incidences of 0.098 for treatment morbidity, 0.024 for treatment death, 0.009 for rupture death, and 0.002 for rupture-related nonfatal poor outcome; the observation arm had no treatment-attributable events, with median incidences of 0.091 for rupture death and 0.019 for rupture-related nonfatal poor outcome. The unweighted endpoint counts each adverse first event once regardless of severity, duration, or reversibility, whereas the QALY analysis distinguishes their health consequences over time. Under the reference QALY configuration, median ΔQALY was + 0.055 (95% uncertainty interval −0.115 to +0.267), although the interval spanned zero. Full component-specific results are reported in S3 Table.

For the disposition-based panels the total rupture first-event incidence is reported separately as a descriptive quantity: the Dasenbrock occupancy trajectory does not identify which ruptures were fatal, so rupture death and rupture-related nonfatal poor outcome are reported as not identifiable rather than as zero.

### Concordance with the event-based endpoint

Comparing the direction of the severity-weighted difference with that of the previously reported unweighted cumulative-incidence difference (30 comparisons, since the Ma panels map to two legacy marginals) gave 21 concordant and 9 discordant comparisons. All observed discordance was directionally asymmetric: panels favoring observation under the unweighted event endpoint shifted toward coiling under the severity-weighted QALY endpoint, whereas no panel shifted in the opposite direction. This pattern is consistent with the reviewer’s proposed mechanism—namely, that equal counting of front-loaded treatment morbidity can disadvantage intervention. Eight of the nine discordant comparisons had QALY uncertainty intervals spanning zero; only the Goldberg 85M comparison excluded zero.

## Structural sensitivity analyses

### Recovery trajectories

Across the reference configuration and the three restricted recovery settings, the assumed permanent fraction of treatment morbidity influenced the QALY difference far more than the assumed duration of temporary morbidity. Within the A5 grid specified in Protocol Amendment v1.5, reducing the permanent fraction from 50% to 25% shifted median ΔQALY by approximately eightfold more than shortening temporary morbidity from 6 to 3 months (median shift 0.041 versus 0.005). Among the reference and three restricted recovery settings, the 50% permanent / 6-month reference was the least favorable to coiling in all 18 mRS panels. Across the full amendment-specified A5 structural envelope, however, the 100% permanent persistence boundary was more conservative still. Median direction changed in 3 of 18 panels across the restricted settings, and in 8 of 18 when the persistence boundary is included. Full panel-level intervals are reported in S4 Table.

### Residual post-treatment rupture risk

Median ΔQALY decreased monotonically with increasing residual rupture risk in all 18 mRS panels. Median direction was unchanged across the 0%, 5%, 10% and 20% grid in 16 of 18 panels. In the Ma 80M panel, the median ΔQALY favored coiling at residual-risk levels of 0% and 5%, but favored observation at 10% and 20%, indicating a sign-change interval between 5% and 10%; the Catapano 90F panel changed direction between 10% and 20%. The amendment-specified 10% reference assumption lies immediately on the observation-favoring side of the Ma 80M crossover interval. Because the 95% uncertainty interval included zero at every residual-risk level in both panels, the result indicates sensitivity to an empirically uncertain structural parameter rather than a definitive reversal in treatment preference. Full panel-level intervals are reported in S5 Table.

### Coiling morbidity

Median ΔQALY increased monotonically as the coiling-morbidity probability was reduced from the 9.8% anchor to 5% and 3%, in all 18 mRS panels. Median direction was unchanged in 14 of 18 panels. In the Ma 80M, Ma 85F and Catapano 90M panels the median direction changed between 9.8% and 5%, and in the Ma 85M panel between 5% and 3%. Reducing the anchor from 9.8% to 3% produced the largest median shift across the amendment-specified ranges examined (median +0.067, range +0.041 to +0.104); the three structural axes span different perturbation ranges and are not directly comparable in magnitude. In all four panels the 95% interval included zero at every morbidity level. Full panel-level intervals are reported in S6 Table.

### Where the sensitivities coincide

Considering the three executed structural axes together, median direction was insensitive to all of them in 13 of 18 mRS panels. One panel (Ma 80M) changed direction on all three axes, two (Ma 85F, Catapano 90M) on two axes, and two (Ma 85M, Catapano 90F) on one. Sign sensitivity was concentrated in panels with reference ΔQALY estimates near zero and substantial uncertainty; all five panels that changed median direction across the executed structural sensitivities had reference 95% intervals spanning zero. Conversely, all eight mRS panels whose reference uncertainty intervals excluded zero retained their median direction across all three executed structural axes (Table 5).

**Table 5:** Median ΔQALY by structural assumption, modified Rankin-based panels. ΔQALY is coiling minus observation; positive favors coiling. Each block varies one assumption while holding the others at the reference configuration. Bold marks a median direction opposite to that panel’s reference value.

**Recovery from treatment morbidity (permanent fraction / temporary duration)**					
**Panel**	**25% / 3 mo**	**25% / 6 mo**	**50% / 6 mo (reference)**	**50% / 3 mo**	**100% permanent**
**Ma — primary mRS outcome reference**					
80, male	**+0.054**	**+0.048**	-0.006	-0.002	-0.113
80, female	+0.128	+0.122	+0.055	+0.059	**-0.079**
85, male	-0.014	-0.020	-0.055	-0.051	-0.126
85, female	**+0.020**	**+0.014**	-0.031	-0.027	-0.120
90, male	-0.040	-0.046	-0.068	-0.064	-0.112
90, female	-0.030	-0.036	-0.064	-0.060	-0.120
**Catapano — treated, mixed severity**					
80, male	+0.225	+0.218	+0.165	+0.169	+0.058
80, female	+0.358	+0.352	+0.285	+0.289	+0.152
85, male	+0.083	+0.077	+0.041	+0.045	**-0.029**
85, female	+0.152	+0.146	+0.101	+0.105	+0.011
90, male	**+0.012**	**+0.007**	-0.015	-0.012	-0.060
90, female	+0.038	+0.033	+0.005	+0.008	**-0.052**
**Goldberg — poor-grade**					
80, male	+0.334	+0.328	+0.274	+0.278	+0.167
80, female	+0.515	+0.508	+0.441	+0.446	+0.308
85, male	+0.143	+0.137	+0.102	+0.106	+0.031
85, female	+0.237	+0.231	+0.186	+0.190	+0.097
90, male	+0.043	+0.038	+0.016	+0.020	**-0.028**
90, female	+0.082	+0.077	+0.049	+0.052	**-0.008**
Median direction changed in 3 of 18 panels across the reference and the three restricted settings, and in 8 of 18 when the 100% permanent persistence boundary is included. The persistence boundary is a continuity limit, not one of the restricted recovery settings.					
**Residual post-treatment rupture risk, as a fraction of the natural-history rate**					
**Panel**	**0%**	**5%**	**10% (reference)**	**20%**	
**Ma — primary mRS outcome reference**					
80, male	**+0.022**	**+0.008**	-0.006	-0.034	
80, female	+0.097	+0.076	+0.055	+0.013	
85, male	-0.042	-0.049	-0.055	-0.069	
85, female	-0.010	-0.020	-0.031	-0.051	
90, male	-0.062	-0.065	-0.068	-0.073	
90, female	-0.055	-0.060	-0.064	-0.073	
**Catapano — treated, mixed severity**					
80, male	+0.214	+0.189	+0.165	+0.117	
80, female	+0.356	+0.320	+0.285	+0.216	
85, male	+0.066	+0.054	+0.041	+0.017	
85, female	+0.137	+0.119	+0.101	+0.065	
90, male	-0.004	-0.010	-0.015	-0.027	
90, female	+0.021	+0.013	+0.005	**-0.012**	
**Goldberg — poor-grade**					
80, male	+0.335	+0.305	+0.274	+0.213	
80, female	+0.531	+0.486	+0.441	+0.355	
85, male	+0.133	+0.118	+0.102	+0.071	
85, female	+0.233	+0.209	+0.186	+0.141	
90, male	+0.031	+0.023	+0.016	+0.001	
90, female	+0.070	+0.059	+0.049	+0.027	
Median ΔQALY decreased monotonically with increasing residual risk in all 18 panels. Direction was unchanged in 16 of 18. 0% is the most coiling-favorable structural boundary of this grid and 20% the least.					
**Periprocedural coiling morbidity**					
**Panel**	**9.8% (reference anchor)**	**5%**		**3%**	
**Ma — primary mRS outcome reference**					
80, male	-0.006	**+0.055**		**+0.080**	
80, female	+0.055	+0.129		+0.159	
85, male	-0.055	-0.013		**+0.005**	
85, female	-0.031	**+0.021**		**+0.043**	
90, male	-0.068	-0.039		-0.027	
90, female	-0.064	-0.029		-0.015	
**Catapano — treated, mixed severity**					
80, male	+0.165	+0.226		+0.251	
80, female	+0.285	+0.359		+0.390	
85, male	+0.041	+0.084		+0.101	
85, female	+0.101	+0.153		+0.174	
90, male	-0.015	**+0.013**		**+0.025**	
90, female	+0.005	+0.039		+0.054	
**Goldberg — poor-grade**					
80, male	+0.274	+0.335		+0.360	
80, female	+0.441	+0.515		+0.546	
85, male	+0.102	+0.144		+0.162	
85, female	+0.186	+0.238		+0.259	
90, male	+0.016	+0.045		+0.057	
90, female	+0.049	+0.083		+0.098	
Median ΔQALY increased monotonically as the anchor was lowered, in all 18 panels. Direction was unchanged in 14 of 18.					

Footnotes: (1) The disposition-based family is not included because the registered structural sensitivity layers apply to the modified Rankin-based panels only. (2) Values are medians of 10,000 probabilistic iterations, discounted at 3% annually. Uncertainty intervals are reported per cell in S4, S5 and S6 Tables. In all 21 panel–axis cells whose median direction differs from that panel’s reference value, the 95% uncertainty interval includes zero, without exception. (3) Each block varies one assumption at a time; the blocks are not crossed, and the three axes span different perturbation ranges, so their effect sizes are not directly comparable. (4) The recovery reference setting (50% permanent, 6-month temporary duration) is an operational choice specified in Protocol Amendment v1.5, not an evidence-derived central estimate.

## Discussion

### Principal findings

The principal finding of the revised analysis is not a reversal from observation to intervention, but a narrowing of what can be inferred from the original unweighted endpoint. Once severity and duration were incorporated through quality-adjusted life years, several panels that had favored observation under the cumulative-incidence endpoint shifted toward coiling, whereas no panel shifted in the opposite direction: of 30 direction comparisons, 21 were concordant and 9 discordant, and all nine discordant comparisons ran in the same direction. This asymmetry is consistent with the mechanism raised in review—that counting front-loaded, potentially transient procedural morbidity as equivalent to permanent disability or death disadvantages intervention—and the component-level results show it concretely. In the Ma 80F panel, the coiling arm carried median incidences of 0.098 for treatment morbidity, 0.024 for treatment death, 0.009 for rupture death, and 0.002 for rupture-related nonfatal poor outcome, whereas the corresponding observation-arm incidences were 0, 0, 0.091, and 0.019. The unweighted endpoint counts each first adverse event once regardless of severity, duration, or reversibility. Eight of the nine discordant comparisons nevertheless had quality-adjusted uncertainty intervals spanning zero. The revision therefore shows that the earlier observation-favoring inference was partly endpoint-dependent; it does not establish the opposite treatment preference.

### Relation to prior literature and what this analysis adds

Prior decision-analytic models for elderly UIA have suggested that preventive treatment loses incremental benefit at advanced ages, but the literature has not systematically examined how this conclusion depends on the assumed post-rupture outcome distribution. Brinjikji and colleagues’ analysis of the National Inpatient Sample [1] provided the periprocedural complication anchors for elderly clipping and coiling cohorts used in the present model, but did not extend to lifetime decision modeling. The Catapano cohort [2] reported treated octogenarian aSAH outcomes with mRS > 2 in 90.7%, while Goldberg and colleagues [4] reported poor-grade ≥ 80–90 mRS 4–6 in 90% at 6–12 month follow-up. Ma and colleagues [5] reported all-grade ≥ 80 aSAH outcomes with markedly different magnitudes (40% mortality, 47% mRS ≥ 3) in a treated cohort with longer follow-up. The natural-history rupture component was anchored to the UCAS-derived elderly cohort reported by Hishikawa and colleagues [13]. In the present model, however, the dominant source of dispersion was not rupture-risk uncertainty alone, but the interaction between competing mortality and the definition of post-rupture poor outcome. These sources provide necessary but non-exchangeable inputs: periprocedural risk, rupture risk, and post-rupture outcome are each anchored to different populations, timepoints, and outcome definitions.

Contemporary flow-diverter treatment also involves device- and anatomy-specific planning that is not represented by simple coiling risk estimates. Briganti et al. [17] evaluated virtual stent deployment as an aid to device sizing and procedural planning, illustrating the technical specificity of this treatment class. Accordingly, the SAC/flow-diversion arm in the present model should be interpreted only as a structural proxy, not as a calibrated estimate of octogenarian outcomes.

This analysis moves beyond the general observation that preventive treatment loses incremental benefit at advanced ages by mapping the modeled scenarios in which that conclusion does or does not hold. Four features support this clinical reading. First, by treating each post-rupture outcome anchor as a parallel scenario rather than averaging across heterogeneous sources, the model makes the outcome-scenario dependence of the model-inferred preference explicit and quantifies that dependence across age and sex panels. Second, the TES process explicitly distinguishes well-supported anchors from weaker scenario constructs, including the SAC/FD proxy assumption, the unanchored VAC stress test, and the Goldberg poor-grade-only caveat. Third, continuous panel-level estimates and uncertainty intervals are reported as the primary outputs, with threshold-zone classification retained only as a secondary interpretive summary. Fourth, parallel reporting of the original cumulative-incidence and severity-weighted quality-adjusted endpoints makes the dependence of model-inferred preference on endpoint definition explicit.

The primary endpoint was revised before submission from a terminal-state occupancy metric to the undiscounted lifetime cumulative incidence of entry into aneurysm- or treatment-attributable states (Protocol Amendment v1.4). Because nonfatal disability states are progressively absorbed into competing mortality over a lifetime horizon, an occupancy metric does not retain nonfatal events and approximates aneurysm- or treatment-attributable mortality alone. By contrast, cumulative incidence retains each event once experienced, a distinction that most affects the front-loaded periprocedural morbidity of the intervention arms. Alongside the outcome-anchor heterogeneity described above, this endpoint sensitivity shows that model-inferred treatment preference in elderly competing-risk decision problems can be materially altered by methodological choices that may otherwise appear interchangeable. This finding supports explicit endpoint auditing and parallel reporting of structural uncertainty.

### Structural sensitivity and parameter dependence

Structural sensitivity was concentrated in panels whose reference quality-adjusted estimates were close to zero and whose uncertainty intervals already spanned zero. For this cross-axis comparison, the recovery axis comprised the reference configuration and the three restricted recovery settings; the 100% permanent persistence boundary was considered separately. Across the three executed structural axes—recovery from treatment morbidity, residual post-treatment rupture risk, and the periprocedural coiling-morbidity anchor—the median direction was unchanged in 13 of 18 modified Rankin-based panels, and all eight panels whose reference intervals excluded zero retained their direction on every axis. These eight comprised both age-90 Ma panels, which favored observation, both age-80 Catapano panels, which favored coiling, and the four age-80 and age-85 Goldberg panels, which also favored coiling. Sign changes were confined to panels with relatively small reference estimates and uncertainty intervals spanning zero. One of them, Ma 80M, changed direction on all three axes; it is an illustrative near-indifference panel rather than an identified subgroup, and its uncertainty interval spanned zero at every setting examined. Within the recovery grid, the assumed permanent fraction of treatment morbidity mattered considerably more than the assumed duration of temporary morbidity, and the 100% permanent persistence boundary is reported separately as a continuity limit rather than as one of the restricted recovery settings.

Two of the model’s inputs that the review identified as weakly grounded were varied directly, and a third was not. Lowering nonfatal coiling morbidity from the historical 9.8% anchor to 5% changed the median direction in three panels, and a further reduction to 3% changed a fourth, without excluding zero in any of those sign-changing comparisons; the analysis quantifies how far the conclusion depends on a treatment-risk estimate drawn from 2001–2008 administrative data, but does not replace that estimate with a contemporary one. Varying the residual post-treatment rupture risk across 0%, 5%, 10% and 20% left the median direction unchanged in 16 of 18 panels; in the Ma 80M panel the direction changed between 5% and 10%, placing the assumed 10% value immediately on the observation-favoring side of that interval, and in the Catapano 90F panel between 10% and 20%. This is a sensitivity analysis, not a calibration: no octogenarian-specific empirical calibration for this residual-rate assumption that is directly applicable to patients aged 80 years and older was identified, and the 10% figure remains a structural assumption whose influence is now quantified rather than an estimated quantity. In contrast, periprocedural coiling mortality was not re-anchored and no mortality sensitivity was performed; the submitted 2.4% estimate therefore remains an unresolved historical limitation of both analyses.

### Clinical interpretation

These findings argue against interpreting either the original cumulative-incidence analysis or the revised quality-adjusted analysis as establishing a universal treatment preference for octogenarians with an unruptured aneurysm. The model stratifies only by index age, sex and post-rupture outcome scenario; it does not represent aneurysm size, location, morphology or growth, previous subarachnoid hemorrhage, frailty, cognitive status, or comorbidity, each of which is central to rupture risk and to procedural risk in this age group. What the analysis can support is a statement about where a modeled preference is stable and where it is not: it is stable at the extremes of the panel set under the conditions described above, and unstable in the panels near indifference, where it moves with the endpoint definition, the recovery assumption, the residual-risk assumption and the procedural-risk anchor. The more defensible conclusion is therefore that modeled treatment preference in this population is conditional on endpoint definition, recovery assumptions, residual rupture risk, and procedural-risk estimates, with the greatest instability occurring in panels already close to indifference. Individual treatment decisions in patients aged 80 years and older require aneurysm- and patient-level information that this model does not contain.

### Outcome anchor mismatch as a structural finding

A central methodological observation from the present analysis is that no single anchor in the available evidence base simultaneously satisfies all of the protocol-primary criteria (all-grade ≥ 80 cohort, mRS 4–6 cutoff, long-term follow-up, large N). The Goldberg anchor (Scenario C) provides the closest match to the mRS 4–6 cutoff but is restricted to poor-grade (WFNS IV-V) patients, includes 80% conservative management, and has only 20 patients. The Ma anchor (Scenarios E and F) provides the all-grade ≥ 80 cohort with long-term follow-up but at broader cutoffs (mortality only, mRS ≥ 3). The Catapano anchor (Scenario A) provides a treated octogenarian cohort but at an even broader cutoff (mRS > 2) and may overestimate poor outcome relative to the protocol-primary mRS 4–6 target. The Dasenbrock anchor (Scenario B) is a disposition-based proxy in an all-grade Medicare population rather than an mRS-based outcome.

This anchor mismatch is not a fixable parameterization choice; it is a structural feature of the present evidence base for octogenarian aSAH outcomes. The implication for the present model is that the dispersion across Scenarios A-F is not merely sampling variation but reflects substantive differences in how the available cohorts define and measure poor outcome. Future prospective cohorts of all-grade octogenarian aSAH with mRS 4–6 ascertainment at 6–12 month follow-up would materially strengthen the evidence base for this decision question.

### Sensitivity behavior under the original cumulative-incidence endpoint

Under the original cumulative-incidence endpoint, the 3% discounted analysis attenuated coiling preference in every coiling-favored panel, the VAC-High stress test eliminated the coiling-favored signal, and the SAC/FD effective-sample-size analysis had only a minor effect on the secondary observation-versus-SAC/FD comparison. The separate post hoc robustness analyses were more sensitive to fixed annual rupture risk and hypothetical coiling morbidity, as detailed in S1 Table. These findings describe sensitivity behavior of the original unweighted endpoint and are not used to characterize the severity-weighted analysis.

### Generalizability

The present model was parameterized for the Korean octogenarian population using KOSIS 2024 life-table competing mortality. The framework is country-portable in structure: substituting an alternative national life table for KOSIS would allow analogous analyses in settings where the Brinjikji periprocedural anchors and the post-rupture outcome scenarios remain applicable. Both periprocedural anchors and outcome scenarios were drawn from primarily US and European cohorts; their applicability to non-Korean Asian populations would benefit from regional validation. The findings should be considered to apply to the modeled Korean cohort under the specified assumptions, and any extrapolation to other settings requires explicit reconsideration of the periprocedural and outcome parameters.

## Conclusions

In this decision-analytic model of unruptured intracranial aneurysm management in patients aged 80 years and older, the observation-favoring pattern seen with the original unweighted cumulative-incidence endpoint was not invariant to endpoint definition. Incorporating severity and duration through a quality-adjusted analysis shifted several observation-favoring comparisons toward coiling, although most discordant estimates remained uncertain. Structural sensitivity to treatment-morbidity recovery, residual post-treatment rupture risk, and the historical coiling-morbidity anchor was concentrated in panels whose reference quality-adjusted differences were already close to zero, whereas panels with reference uncertainty intervals excluding zero retained their direction across the executed structural analyses. These findings do not establish a universal preference for either observation or coiling. Rather, modeled treatment preference is conditional on endpoint definition and key structural assumptions, and individual treatment decisions require aneurysm- and patient-level characteristics that were not represented in the model.

### Limitations

The present analysis has nine principal limitations, described below.

#### L1. Outcome scenario heterogeneity.

The five post-rupture outcome scenarios (A, B, C, E, F) differ in cohort grade, outcome cutoff, and timepoint of ascertainment. The model-inferred preference is highly sensitive to which scenario is used as the operative assumption (the probability that coiling was favored over observation ranged from 0.000 to 0.984 across panels). The present analysis reports results in parallel across all scenarios rather than selecting one as primary; this preserves transparency but does not resolve the question of which scenario most closely reflects the protocol-primary target (all-grade ≥ 80 mRS 4–6 at 6–12 month follow-up).

#### L2. Unanchored comparator constructs: SAC/FD and vascular access complexity.

The SAC/FD scenario uses the coiling mean as its proxy point estimate with a downweighted effective sample size (50 in the base case and 100 in sensitivity), because the targeted evidence synthesis identified no direct peer-reviewed clinical-burden evidence for patients aged 80 years and older. SAC/FD outputs therefore describe model behavior under a proxy assumption rather than calibrated estimates of SAC/FD performance in octogenarian practice.

The vascular access complexity modifier was originally planned as a quantitatively anchored parameter but was demoted to an unanchored stress test when no calibrated effect estimate for the ≥ 80 endovascular cohort could be identified. The base case sets VAC to 1.0; the VAC-High sensitivity applies a lognormal multiplier with a median of 2.0 and nominal bounds of 1.5–2.5 used to define the log-scale dispersion. This range was chosen for illustration rather than estimated from a clinical cohort.

Neither construct provides a calibrated estimate for octogenarian practice. Both were retained as secondary structural scenarios and did not determine the principal observation-versus-coiling comparison.

#### L3. Korean life-table competing mortality treated deterministically.

The KOSIS qx values were treated as deterministic point estimates in the base case PSA. Variation in competing mortality between regional cohorts and over time was not propagated through the PSA. An alternative life-table sampling sensitivity was prespecified but has not been executed in the present analysis.

#### L4. Residual rupture risk after treatment fixed at 10%.

The model assumes the post-treatment residual annual rupture risk is 10% of the natural-history rate. This single-value assumption was not explicitly sampled in the original PSA. An amendment-specified sensitivity analysis varying this fraction across 0%, 5%, 10% and 20% was executed under the severity-weighted endpoint. Median direction was unchanged in 16 of 18 mRS-based panels. In the Ma 80M panel the median difference favored coiling at 0% and 5% and favored observation at 10% and 20%, indicating a sign-change interval between 5% and 10%, and the Catapano 90F panel changed direction between 10% and 20%; in both panels the 95% uncertainty interval included zero at every level. The 10% assumption therefore remains an uncalibrated structural choice rather than an empirically estimated quantity, and the sensitivity analysis quantifies how far the conclusion depends on it. It does not supply a calibrated value: no octogenarian-specific empirical basis for the residual rate was identified in the targeted evidence synthesis.

#### L5. Lifetime horizon and Korean setting.

The model uses a lifetime horizon extending beyond age 100 and is parameterized for the Korean octogenarian population. Generalizability to other settings requires substitution of the competing-mortality input. The lifetime horizon assumption is standard for decision-analytic models but extends well beyond the follow-up duration of any of the anchor cohorts (the longest follow-up among anchor cohorts was Ma et al. [5] at median 17 months).

#### L6. Primary endpoint revision and base-case discounting.

The primary endpoint was revised before submission. The originally implemented metric measured occupancy of aneurysm- or treatment-attributable states at the end of the lifetime horizon; because the non-absorbing disability states (treatment morbidity and rupture poor outcome) transitioned to competing mortality over time, that metric did not retain nonfatal events and approximated cumulative aneurysm/treatment mortality. We adopted the undiscounted lifetime cumulative incidence as the primary endpoint to retain each event once experienced, consistent with the intended composite-event interpretation (Protocol Amendment v1.4); the superseded terminal-occupancy analysis is retained in the project archive for transparency. The base case uses a 0% annual discount rate, which is defensible for elderly-population lifetime decision analyses but is not the CHEERS 2022 default [14]; the 3% discounted cumulative-incidence analysis is reported as the key sensitivity and uses the same cumulative-incidence metric as the base case.

The severity-weighted analysis added in revision does not replace this endpoint; both are reported. The two use different discounting conventions: the cumulative-incidence analysis is undiscounted in the base case with a 3% discounted analysis as its key sensitivity, whereas the quality-adjusted-life-year analysis uses a 3% annual discount rate as its reference, following the registered amendment. Discounted and undiscounted quantities are therefore not directly comparable across the two endpoints, and no comparison of that kind is made.

The registered discount-rate sensitivity for the severity-weighted endpoint, which would have examined 0% and 4.5% alongside the 3% reference across 48 evaluations, was not executed; that omission is recorded in the deviation statement. The robustness of the reference quality-adjusted findings to alternative discount rates was therefore not assessed.

#### L7. Severity weighting and the assumptions it introduces.

The cumulative-incidence endpoint counts nonfatal periprocedural morbidity and rupture-related poor outcome as equivalent single events and does not weight events by severity or by time spent in a disabled state. The severity-weighted analysis added in revision addresses that limitation directly, but introduces assumptions of its own. The utility values are fixed point estimates rather than sampled quantities, and utility uncertainty was therefore not propagated probabilistically. The registered plan treated utility-mapping choice as a structural sensitivity, but the alternative-mapping layer was not executed. Grouped modified Rankin outcomes are represented by band-level utilities, and the registered within-bin utility sensitivity was not executed. Likewise, the disposition-based panels rely on a crosswalk from discharge disposition to utility that is a reasoned proxy rather than a validated equivalence, and the registered institutional-utility sensitivity was not executed. Accordingly, the reported quality-adjusted uncertainty intervals do not encompass these utility-specification uncertainties. The recovery parameters—the permanent fraction of treatment morbidity and the duration of temporary morbidity—are an illustrative structural envelope, not empirical estimates, and the reference setting of 50% permanent with a 6-month temporary-morbidity duration is an operational choice; among the reference and three restricted recovery settings it was the least favorable to coiling in all 18 mRS panels, while the 100% permanent persistence boundary was more conservative still.

#### L8. Periprocedural treatment-risk anchor era.

The periprocedural treatment-risk anchors were drawn from Brinjikji and colleagues’ National Inpatient Sample analysis of 2001–2008 admissions [1]. These estimates may not fully reflect contemporary endovascular devices, operator experience, anesthesia practice, or periprocedural care; if current coiling morbidity is substantially lower than the anchored estimate used here, the model may understate the range of scenarios in which preventive coiling could be favored. The post hoc coiling-morbidity threshold analysis (S1 Table) varied only nonfatal coiling morbidity and did not re-anchor coiling mortality, case selection, device era, anesthesia practice, or institutional operator effects; it should therefore be interpreted as a hypothetical threshold analysis rather than a contemporary-treatment re-estimation.

Under the severity-weighted endpoint, an amendment-specified sensitivity analysis repeated the analysis with the coiling-morbidity probability set to 5% and 3% against the 9.8% anchor. Median direction was unchanged in 14 of 18 mRS-based panels; the Ma 80M, Ma 85F and Catapano 90M panels changed direction between 9.8% and 5%, and the Ma 85M panel between 5% and 3%, with the 95% uncertainty interval including zero at every level in all four. This quantifies the dependence of the conclusion on the historical morbidity anchor; it does not replace that anchor with a contemporary estimate.

Periprocedural coiling mortality was not varied. The targeted evidence synthesis identified no contemporary, adequately powered estimate restricted to patients aged 80 years or older with an outcome definition compatible with immediate periprocedural mortality. Accordingly, the submitted 2.4% estimate was retained, and the mortality anchor remains a limitation that this revision does not resolve.

#### L9. Aneurysm-level rupture-risk determinants were not modeled.

The model represents an average-risk unruptured aneurysm parameterized by an assumed annual rupture probability; it does not incorporate aneurysm-level determinants of rupture risk such as size, location, morphology (irregularity or daughter sac), multiplicity, documented growth, prior subarachnoid hemorrhage, or family history, nor composite risk scores (e.g., PHASES, UCAS/UIATS). The fixed annual rupture-risk scenarios examined in the post hoc analysis (ranging from 0.5% to 5.0%) span a range that partly reflects these determinants but do not represent specific aneurysm phenotypes. The present model should therefore be interpreted as a parameterized decision framework, not as an aneurysm morphology-specific treatment calculator, and its outputs should not be applied to an individual aneurysm without separate consideration of these factors.

## Supporting information

S1 Table Post hoc robustness analyses. Number of coiling-favored panels and panel-level probability that coiling was favored over observation, under fixed annual rupture-risk scenarios (0.5%, 1.0%, 2.5%, 5.0%) and coiling-morbidity thresholds (5%, 3%).
(DOCX)

S2 Table Panels assigned to zones T1–T2 under the original cumulative-incidence endpoint. (DOCX)

S3 Table Component-specific first-event cumulative incidences by panel and strategy under the reference QALY configuration. (DOCX)

S4 Table Recovery-trajectory sensitivity of the severity-weighted analysis. (DOCX)

S5 Table Residual post-treatment rupture-risk sensitivity of the severity-weighted analysis. (DOCX)

S6 Table Periprocedural coiling-morbidity sensitivity of the severity-weighted analysis. (DOCX)

S1 File Reproducible analysis code and per-panel results for the post hoc robustness analyses (Python script and results table). (ZIP)

S2 File Minimized KOSIS model-input workbook. An anonymized model-cycle- and panel-indexed record of competing-mortality probabilities prepared for the journal-facing supporting data file; the exact age-indexed inputs used by the model are preserved in the OSF reproducibility archive.
(XLSX)
