# Supplementary material for: Observation versus coiling for unruptured intracranial aneurysms in octogenarians: A decision-analytic modeling study
Source: PLoS One. 2026 Sep 1;21(9):e0357374. doi: 10.1371/journal.pone.0357374 (PMC13533429; doi:10.1371/journal.pone.0357374)
Supplement: Supplementary file 1 [file pone.0357374.s001.docx]

**Supplementary Table S1. Post hoc robustness analyses**

*Number of coiling-favored panels and panel-level classification under fixed annual rupture-risk scenarios (0.5%, 1.0%, 2.5%, 5.0%) and coiling-morbidity thresholds (5%, 3%), compared with the base case. A panel was classified as coiling-favored when it fell in threshold zone T1 or T2. All analyses used the same competing-risk engine, ±0.01 equipoise band, and threshold-zone classification as the base case; the base-case probabilistic parameter structure was preserved and only the indicated parameter was overridden in each analysis.*

**Panel A. Summary**

| **Analysis** | **Coiling-favored (n/30)** | **Age 80** | **Age 85** | **Age 90** |
| --- | --- | --- | --- | --- |
| **Base case** | **7/30** | **5** | **2** | **0** |
| Rupture 0.5% | 0/30 | 0 | 0 | 0 |
| Rupture 1.0% | 0/30 | 0 | 0 | 0 |
| Rupture 2.5% | 7/30 | 5 | 2 | 0 |
| Rupture 5.0% | 24/30 | 10 | 9 | 5 |
| Coil morb 5% | 20/30 | 9 | 7 | 4 |
| Coil morb 3% | 24/30 | 10 | 9 | 5 |

*Resistant subset: the same six panels — 85M-E, 90M-B, 90M-E, 90M-F, 90F-E, 90F-F (predominantly index-age-90 and the all-grade post-rupture outcome Scenarios E and F) — remained non–coiling-favored under both the most coiling-favorable assumptions tested (5.0% annual rupture risk and 3% coiling morbidity).*

**Panel B. Panel-level probability that coiling was favored over observation**

| **Panel** | **Base case** | **Rupture 0.5%** | **Rupture 1.0%** | **Rupture 2.5%** | **Rupture 5.0%** | **Coil morb 5%** | **Coil morb 3%** |
| --- | --- | --- | --- | --- | --- | --- | --- |
| **80M-A** | **0.851** | 0.000 | 0.000 | **0.975** | **1.000** | **1.000** | **1.000** |
| **80M-B** | 0.264 | 0.000 | 0.000 | 0.101 | **1.000** | **0.968** | **0.999** |
| **80M-C** | **0.837** | 0.000 | 0.000 | **0.948** | **1.000** | **0.999** | **1.000** |
| **80M-E** | 0.012 | 0.000 | 0.000 | 0.000 | **0.658** | 0.310 | **0.707** |
| **80M-F** | 0.045 | 0.000 | 0.000 | 0.008 | **0.903** | **0.576** | **0.897** |
| **80F-A** | **0.984** | 0.000 | 0.000 | **1.000** | **1.000** | **1.000** | **1.000** |
| **80F-B** | **0.695** | 0.000 | 0.000 | **0.863** | **1.000** | **0.999** | **1.000** |
| **80F-C** | **0.980** | 0.000 | 0.000 | **0.999** | **1.000** | **1.000** | **1.000** |
| **80F-E** | 0.081 | 0.000 | 0.000 | 0.027 | **0.908** | **0.639** | **0.905** |
| **80F-F** | 0.220 | 0.000 | 0.000 | 0.148 | **0.988** | **0.863** | **0.982** |
| **85M-A** | 0.245 | 0.000 | 0.000 | 0.095 | **1.000** | **0.958** | **0.998** |
| **85M-B** | 0.009 | 0.000 | 0.000 | 0.000 | **0.985** | **0.501** | **0.925** |
| **85M-C** | 0.238 | 0.000 | 0.000 | 0.098 | **1.000** | **0.950** | **0.998** |
| **85M-E** | 0.000 | 0.000 | 0.000 | 0.000 | 0.102 | 0.031 | 0.230 |
| **85M-F** | 0.001 | 0.000 | 0.000 | 0.000 | **0.333** | 0.108 | **0.469** |
| **85F-A** | **0.663** | 0.000 | 0.000 | **0.792** | **1.000** | **0.998** | **1.000** |
| **85F-B** | 0.105 | 0.000 | 0.000 | 0.007 | **1.000** | **0.881** | **0.996** |
| **85F-C** | **0.649** | 0.000 | 0.000 | **0.752** | **1.000** | **0.996** | **1.000** |
| **85F-E** | 0.003 | 0.000 | 0.000 | 0.000 | **0.434** | 0.161 | **0.527** |
| **85F-F** | 0.014 | 0.000 | 0.000 | 0.000 | **0.769** | **0.375** | **0.785** |
| **90M-A** | 0.007 | 0.000 | 0.000 | 0.000 | **0.952** | **0.442** | **0.894** |
| **90M-B** | 0.000 | 0.000 | 0.000 | 0.000 | 0.062 | 0.028 | 0.344 |
| **90M-C** | 0.007 | 0.000 | 0.000 | 0.000 | **0.922** | **0.435** | **0.885** |
| **90M-E** | 0.000 | 0.000 | 0.000 | 0.000 | 0.000 | 0.000 | 0.016 |
| **90M-F** | 0.000 | 0.000 | 0.000 | 0.000 | 0.005 | 0.003 | 0.065 |
| **90F-A** | 0.069 | 0.000 | 0.000 | 0.003 | **1.000** | **0.812** | **0.987** |
| **90F-B** | 0.000 | 0.000 | 0.000 | 0.000 | **0.682** | 0.188 | **0.723** |
| **90F-C** | 0.067 | 0.000 | 0.000 | 0.003 | **0.995** | **0.798** | **0.984** |
| **90F-E** | 0.000 | 0.000 | 0.000 | 0.000 | 0.014 | 0.005 | 0.086 |
| **90F-F** | 0.000 | 0.000 | 0.000 | 0.000 | 0.084 | 0.027 | 0.231 |

*Values are the probability that coiling produced a lower unweighted lifetime cumulative incidence of first aneurysm- or treatment-attributable adverse events than observation, beyond the +0.01 equipoise band, across 10,000 probabilistic iterations per panel. Bold values denote coiling-favored panels (threshold zone T1 or T2). Panels are labeled by index age (80/85/90), sex (M/F), and post-rupture outcome scenario (A, B, C, E, F).*
