# Supplementary material for: Observation versus coiling for unruptured intracranial aneurysms in octogenarians: A decision-analytic modeling study
Source: PLoS One. 2026 Sep 1;21(9):e0357374. doi: 10.1371/journal.pone.0357374 (PMC13533429; doi:10.1371/journal.pone.0357374)
Supplement: Supplementary file 2 [file pone.0357374.s002.docx]

**S2 Table. Panels assigned to zones T1–T2 under the original cumulative-incidence endpoint.**

This listing restates the continuous estimates in Table 2 under the prespecified ±0.01 equipoise band and hierarchical threshold rule; it is provided as a secondary interpretive summary and is not an independent finding.

| **Age** | **Sex** | **Scenario** | **Median Δ (obs−coil)** | **P(coiling) 0%** | **P(coiling) 3% disc.** | **P(obs) 0%** | **Zone** |
| --- | --- | --- | --- | --- | --- | --- | --- |
| 80 | F | A (Catapano, mRS>2) | 0.0733 | 0.984 | 0.896 | 0.002 | T1 |
| 80 | F | B (Dasenbrock disposition) | 0.0230 | 0.695 | 0.342 | 0.086 | T2 |
| 80 | F | C (Goldberg poor-grade mRS 4–6) | 0.0724 | 0.980 | 0.886 | 0.003 | T1 |
| 80 | M | A (Catapano, mRS>2) | 0.0372 | 0.851 | 0.610 | 0.035 | T2 |
| 80 | M | C (Goldberg poor-grade mRS 4–6) | 0.0364 | 0.837 | 0.597 | 0.041 | T2 |
| 85 | F | A (Catapano, mRS>2) | 0.0203 | 0.663 | 0.400 | 0.106 | T2 |
| 85 | F | C (Goldberg poor-grade mRS 4–6) | 0.0196 | 0.649 | 0.397 | 0.118 | T2 |

Δ = difference in lifetime cumulative incidence (observation minus coiling); positive values favor coiling. P(obs), probability that observation was favored. Zones T1–T2 are defined in the main-text Methods.
