# Supplementary material for: Observation versus coiling for unruptured intracranial aneurysms in octogenarians: A decision-analytic modeling study
Source: PLoS One. 2026 Sep 1;21(9):e0357374. doi: 10.1371/journal.pone.0357374 (PMC13533429; doi:10.1371/journal.pone.0357374)
Supplement: Supplementary file 3 [file pone.0357374.s003.docx]

**S3 Table. Component-specific first-event cumulative incidences by panel and strategy. Values are medians with 95% uncertainty intervals from 10,000 probabilistic iterations. The reference QALY configuration uses 3% annual discounting for QALYs; the component incidences themselves are cumulative first-event quantities.**

| **Outcome source** | **Age** | **Sex** | **Strategy** | **Treatment morbidity** | **Treatment death** | **Rupture death** | **Rupture-related nonfatal poor outcome** | **All first ruptures (descriptive)** |
| --- | --- | --- | --- | --- | --- | --- | --- | --- |
| Ma — primary mRS outcome reference | 80 | M | Observation | 0.000 (0.000 to 0.000) | 0.000 (0.000 to 0.000) | 0.075 (0.044 to 0.119) | 0.016 (0.004 to 0.039) | 0.192 (0.141 to 0.259) |
| Ma — primary mRS outcome reference | 80 | M | Coiling | 0.098 (0.098 to 0.098) | 0.024 (0.015 to 0.036) | 0.007 (0.004 to 0.012) | 0.002 (0.000 to 0.004) | 0.019 (0.013 to 0.027) |
| Ma — primary mRS outcome reference | 80 | F | Observation | 0.000 (0.000 to 0.000) | 0.000 (0.000 to 0.000) | 0.091 (0.054 to 0.144) | 0.019 (0.005 to 0.048) | 0.236 (0.175 to 0.315) |
| Ma — primary mRS outcome reference | 80 | F | Coiling | 0.098 (0.098 to 0.098) | 0.024 (0.015 to 0.036) | 0.009 (0.005 to 0.015) | 0.002 (0.001 to 0.005) | 0.024 (0.017 to 0.033) |
| Ma — primary mRS outcome reference | 85 | M | Observation | 0.000 (0.000 to 0.000) | 0.000 (0.000 to 0.000) | 0.054 (0.031 to 0.086) | 0.011 (0.003 to 0.028) | 0.139 (0.102 to 0.190) |
| Ma — primary mRS outcome reference | 85 | M | Coiling | 0.098 (0.098 to 0.098) | 0.024 (0.015 to 0.036) | 0.005 (0.003 to 0.008) | 0.001 (0.000 to 0.003) | 0.013 (0.009 to 0.019) |
| Ma — primary mRS outcome reference | 85 | F | Observation | 0.000 (0.000 to 0.000) | 0.000 (0.000 to 0.000) | 0.067 (0.038 to 0.105) | 0.014 (0.004 to 0.036) | 0.172 (0.126 to 0.233) |
| Ma — primary mRS outcome reference | 85 | F | Coiling | 0.098 (0.098 to 0.098) | 0.024 (0.015 to 0.036) | 0.006 (0.004 to 0.011) | 0.001 (0.000 to 0.004) | 0.017 (0.012 to 0.023) |
| Ma — primary mRS outcome reference | 90 | M | Observation | 0.000 (0.000 to 0.000) | 0.000 (0.000 to 0.000) | 0.039 (0.023 to 0.062) | 0.008 (0.002 to 0.020) | 0.099 (0.072 to 0.137) |
| Ma — primary mRS outcome reference | 90 | M | Coiling | 0.098 (0.098 to 0.098) | 0.024 (0.015 to 0.036) | 0.004 (0.002 to 0.006) | 0.001 (0.000 to 0.002) | 0.009 (0.007 to 0.013) |
| Ma — primary mRS outcome reference | 90 | F | Observation | 0.000 (0.000 to 0.000) | 0.000 (0.000 to 0.000) | 0.046 (0.027 to 0.074) | 0.010 (0.003 to 0.025) | 0.120 (0.087 to 0.164) |
| Ma — primary mRS outcome reference | 90 | F | Coiling | 0.098 (0.098 to 0.098) | 0.024 (0.015 to 0.036) | 0.004 (0.002 to 0.007) | 0.001 (0.000 to 0.002) | 0.011 (0.008 to 0.016) |
| Catapano — treated, mixed severity | 80 | M | Observation | 0.000 (0.000 to 0.000) | 0.000 (0.000 to 0.000) | 0.062 (0.035 to 0.100) | 0.109 (0.072 to 0.160) | 0.192 (0.141 to 0.259) |
| Catapano — treated, mixed severity | 80 | M | Coiling | 0.098 (0.098 to 0.098) | 0.024 (0.015 to 0.036) | 0.006 (0.003 to 0.010) | 0.011 (0.007 to 0.016) | 0.019 (0.013 to 0.027) |
| Catapano — treated, mixed severity | 80 | F | Observation | 0.000 (0.000 to 0.000) | 0.000 (0.000 to 0.000) | 0.076 (0.043 to 0.122) | 0.134 (0.089 to 0.193) | 0.236 (0.174 to 0.315) |
| Catapano — treated, mixed severity | 80 | F | Coiling | 0.098 (0.098 to 0.098) | 0.024 (0.015 to 0.036) | 0.008 (0.004 to 0.013) | 0.014 (0.009 to 0.020) | 0.024 (0.017 to 0.034) |
| Catapano — treated, mixed severity | 85 | M | Observation | 0.000 (0.000 to 0.000) | 0.000 (0.000 to 0.000) | 0.045 (0.025 to 0.073) | 0.079 (0.052 to 0.117) | 0.139 (0.101 to 0.191) |
| Catapano — treated, mixed severity | 85 | M | Coiling | 0.098 (0.098 to 0.098) | 0.024 (0.015 to 0.036) | 0.004 (0.002 to 0.007) | 0.008 (0.005 to 0.011) | 0.013 (0.009 to 0.019) |
| Catapano — treated, mixed severity | 85 | F | Observation | 0.000 (0.000 to 0.000) | 0.000 (0.000 to 0.000) | 0.056 (0.031 to 0.090) | 0.097 (0.064 to 0.143) | 0.172 (0.125 to 0.233) |
| Catapano — treated, mixed severity | 85 | F | Coiling | 0.098 (0.098 to 0.098) | 0.024 (0.015 to 0.036) | 0.005 (0.003 to 0.009) | 0.009 (0.006 to 0.014) | 0.017 (0.012 to 0.024) |
| Catapano — treated, mixed severity | 90 | M | Observation | 0.000 (0.000 to 0.000) | 0.000 (0.000 to 0.000) | 0.032 (0.018 to 0.053) | 0.056 (0.037 to 0.084) | 0.099 (0.071 to 0.137) |
| Catapano — treated, mixed severity | 90 | M | Coiling | 0.098 (0.098 to 0.098) | 0.024 (0.015 to 0.036) | 0.003 (0.002 to 0.005) | 0.005 (0.003 to 0.008) | 0.009 (0.006 to 0.013) |
| Catapano — treated, mixed severity | 90 | F | Observation | 0.000 (0.000 to 0.000) | 0.000 (0.000 to 0.000) | 0.038 (0.022 to 0.064) | 0.068 (0.044 to 0.101) | 0.120 (0.086 to 0.165) |
| Catapano — treated, mixed severity | 90 | F | Coiling | 0.098 (0.098 to 0.098) | 0.024 (0.015 to 0.036) | 0.004 (0.002 to 0.006) | 0.006 (0.004 to 0.010) | 0.011 (0.008 to 0.016) |
| Goldberg — poor-grade | 80 | M | Observation | 0.000 (0.000 to 0.000) | 0.000 (0.000 to 0.000) | 0.165 (0.116 to 0.231) | 0.002 (0.000 to 0.022) | 0.192 (0.142 to 0.259) |
| Goldberg — poor-grade | 80 | M | Coiling | 0.098 (0.098 to 0.098) | 0.024 (0.015 to 0.036) | 0.016 (0.011 to 0.024) | 0.000 (0.000 to 0.002) | 0.019 (0.014 to 0.027) |
| Goldberg — poor-grade | 80 | F | Observation | 0.000 (0.000 to 0.000) | 0.000 (0.000 to 0.000) | 0.204 (0.142 to 0.281) | 0.003 (0.000 to 0.027) | 0.236 (0.175 to 0.315) |
| Goldberg — poor-grade | 80 | F | Coiling | 0.098 (0.098 to 0.098) | 0.024 (0.015 to 0.036) | 0.021 (0.014 to 0.030) | 0.000 (0.000 to 0.003) | 0.024 (0.017 to 0.034) |
| Goldberg — poor-grade | 85 | M | Observation | 0.000 (0.000 to 0.000) | 0.000 (0.000 to 0.000) | 0.120 (0.082 to 0.169) | 0.002 (0.000 to 0.016) | 0.140 (0.102 to 0.191) |
| Goldberg — poor-grade | 85 | M | Coiling | 0.098 (0.098 to 0.098) | 0.024 (0.015 to 0.036) | 0.011 (0.008 to 0.017) | 0.000 (0.000 to 0.002) | 0.013 (0.010 to 0.019) |
| Goldberg — poor-grade | 85 | F | Observation | 0.000 (0.000 to 0.000) | 0.000 (0.000 to 0.000) | 0.148 (0.102 to 0.206) | 0.002 (0.000 to 0.020) | 0.172 (0.126 to 0.233) |
| Goldberg — poor-grade | 85 | F | Coiling | 0.098 (0.098 to 0.098) | 0.024 (0.015 to 0.036) | 0.014 (0.010 to 0.021) | 0.000 (0.000 to 0.002) | 0.017 (0.012 to 0.024) |
| Goldberg — poor-grade | 90 | M | Observation | 0.000 (0.000 to 0.000) | 0.000 (0.000 to 0.000) | 0.085 (0.058 to 0.121) | 0.001 (0.000 to 0.012) | 0.099 (0.072 to 0.137) |
| Goldberg — poor-grade | 90 | M | Coiling | 0.098 (0.098 to 0.098) | 0.024 (0.015 to 0.036) | 0.008 (0.005 to 0.012) | 0.000 (0.000 to 0.001) | 0.009 (0.007 to 0.013) |
| Goldberg — poor-grade | 90 | F | Observation | 0.000 (0.000 to 0.000) | 0.000 (0.000 to 0.000) | 0.103 (0.071 to 0.146) | 0.001 (0.000 to 0.014) | 0.120 (0.087 to 0.165) |
| Goldberg — poor-grade | 90 | F | Coiling | 0.098 (0.098 to 0.098) | 0.024 (0.015 to 0.036) | 0.010 (0.007 to 0.014) | 0.000 (0.000 to 0.001) | 0.011 (0.008 to 0.016) |
| Dasenbrock — secondary disposition-based outcome family | 80 | M | Observation | 0.000 (0.000 to 0.000) | 0.000 (0.000 to 0.000) | NI | NI | 0.192 (0.141 to 0.258) |
| Dasenbrock — secondary disposition-based outcome family | 80 | M | Coiling | 0.098 (0.098 to 0.098) | 0.023 (0.015 to 0.036) | NI | NI | 0.019 (0.013 to 0.027) |
| Dasenbrock — secondary disposition-based outcome family | 80 | F | Observation | 0.000 (0.000 to 0.000) | 0.000 (0.000 to 0.000) | NI | NI | 0.236 (0.174 to 0.314) |
| Dasenbrock — secondary disposition-based outcome family | 80 | F | Coiling | 0.098 (0.098 to 0.098) | 0.023 (0.015 to 0.036) | NI | NI | 0.024 (0.017 to 0.033) |
| Dasenbrock — secondary disposition-based outcome family | 85 | M | Observation | 0.000 (0.000 to 0.000) | 0.000 (0.000 to 0.000) | NI | NI | 0.140 (0.101 to 0.190) |
| Dasenbrock — secondary disposition-based outcome family | 85 | M | Coiling | 0.098 (0.098 to 0.098) | 0.023 (0.015 to 0.036) | NI | NI | 0.013 (0.009 to 0.019) |
| Dasenbrock — secondary disposition-based outcome family | 85 | F | Observation | 0.000 (0.000 to 0.000) | 0.000 (0.000 to 0.000) | NI | NI | 0.172 (0.125 to 0.232) |
| Dasenbrock — secondary disposition-based outcome family | 85 | F | Coiling | 0.098 (0.098 to 0.098) | 0.023 (0.015 to 0.036) | NI | NI | 0.017 (0.012 to 0.023) |
| Dasenbrock — secondary disposition-based outcome family | 90 | M | Observation | 0.000 (0.000 to 0.000) | 0.000 (0.000 to 0.000) | NI | NI | 0.099 (0.071 to 0.137) |
| Dasenbrock — secondary disposition-based outcome family | 90 | M | Coiling | 0.098 (0.098 to 0.098) | 0.023 (0.015 to 0.036) | NI | NI | 0.009 (0.007 to 0.013) |
| Dasenbrock — secondary disposition-based outcome family | 90 | F | Observation | 0.000 (0.000 to 0.000) | 0.000 (0.000 to 0.000) | NI | NI | 0.120 (0.086 to 0.164) |
| Dasenbrock — secondary disposition-based outcome family | 90 | F | Coiling | 0.098 (0.098 to 0.098) | 0.023 (0.015 to 0.036) | NI | NI | 0.011 (0.008 to 0.016) |

1. Treatment morbidity, treatment death, rupture death, and rupture-related nonfatal poor outcome are the four adverse first-event components used by the original cumulative-incidence endpoint. They are shown separately here; they are not severity weighted in this table.

2. All first ruptures is a descriptive quantity and is not an additional component of the original adverse first-event composite. It includes every first rupture, including ruptures followed by better recovery.

3. In the Dasenbrock disposition-based family, rupture death and rupture-related nonfatal poor outcome are not identifiable (NI) from the source-defined occupancy trajectory and are therefore not reported as zero. The all-first-ruptures total remains identifiable.

4. The disposition-based family is a secondary outcome-definition family and is not pooled with the modified Rankin Scale-based sources.
