# Supplementary material for: Observation versus coiling for unruptured intracranial aneurysms in octogenarians: A decision-analytic modeling study
Source: PLoS One. 2026 Sep 1;21(9):e0357374. doi: 10.1371/journal.pone.0357374 (PMC13533429; doi:10.1371/journal.pone.0357374)
Supplement: Supplementary file 4 [file pone.0357374.s004.docx]

**S4 Table. recovery-trajectory sensitivity. ΔQALY is coiling minus observation; positive values favor coiling. Values are medians with 95% uncertainty intervals and P(ΔQALY > 0) from 10,000 probabilistic iterations, with QALYs discounted at 3% annually.**

| **Outcome source** | **Age** | **Sex** | **Setting** | **Median ΔQALY** | **95% uncertainty interval** | **P(ΔQALY > 0)** |
| --- | --- | --- | --- | --- | --- | --- |
| Ma — primary mRS outcome reference | 80 | M | 25% permanent / 3-month temporary duration | +0.054 | -0.069 to +0.208 | 0.793 |
| Ma — primary mRS outcome reference | 80 | M | 25% permanent / 6-month temporary duration | +0.048 | -0.075 to +0.202 | 0.764 |
| Ma — primary mRS outcome reference | 80 | M | 50% permanent / 6-month temporary duration (reference) | -0.006 | -0.129 to +0.148 | 0.459 |
| Ma — primary mRS outcome reference | 80 | M | 50% permanent / 3-month temporary duration | -0.002 | -0.125 to +0.152 | 0.488 |
| Ma — primary mRS outcome reference | 80 | M | 100% permanent persistence boundary | -0.113 | -0.236 to +0.042 | 0.069 |
| Ma — primary mRS outcome reference | 80 | F | 25% permanent / 3-month temporary duration | +0.128 | -0.042 to +0.340 | 0.929 |
| Ma — primary mRS outcome reference | 80 | F | 25% permanent / 6-month temporary duration | +0.122 | -0.049 to +0.333 | 0.919 |
| Ma — primary mRS outcome reference | 80 | F | 50% permanent / 6-month temporary duration (reference) | +0.055 | -0.115 to +0.267 | 0.731 |
| Ma — primary mRS outcome reference | 80 | F | 50% permanent / 3-month temporary duration | +0.059 | -0.111 to +0.271 | 0.746 |
| Ma — primary mRS outcome reference | 80 | F | 100% permanent persistence boundary | -0.079 | -0.249 to +0.134 | 0.217 |
| Ma — primary mRS outcome reference | 85 | M | 25% permanent / 3-month temporary duration | -0.014 | -0.086 to +0.070 | 0.360 |
| Ma — primary mRS outcome reference | 85 | M | 25% permanent / 6-month temporary duration | -0.020 | -0.092 to +0.064 | 0.310 |
| Ma — primary mRS outcome reference | 85 | M | 50% permanent / 6-month temporary duration (reference) | -0.055 | -0.128 to +0.029 | 0.093 |
| Ma — primary mRS outcome reference | 85 | M | 50% permanent / 3-month temporary duration | -0.051 | -0.124 to +0.033 | 0.111 |
| Ma — primary mRS outcome reference | 85 | M | 100% permanent persistence boundary | -0.126 | -0.198 to -0.041 | 0.003 |
| Ma — primary mRS outcome reference | 85 | F | 25% permanent / 3-month temporary duration | +0.020 | -0.079 to +0.137 | 0.649 |
| Ma — primary mRS outcome reference | 85 | F | 25% permanent / 6-month temporary duration | +0.014 | -0.085 to +0.131 | 0.605 |
| Ma — primary mRS outcome reference | 85 | F | 50% permanent / 6-month temporary duration (reference) | -0.031 | -0.130 to +0.086 | 0.290 |
| Ma — primary mRS outcome reference | 85 | F | 50% permanent / 3-month temporary duration | -0.027 | -0.126 to +0.090 | 0.316 |
| Ma — primary mRS outcome reference | 85 | F | 100% permanent persistence boundary | -0.120 | -0.220 to -0.003 | 0.022 |
| Ma — primary mRS outcome reference | 90 | M | 25% permanent / 3-month temporary duration | -0.040 | -0.082 to +0.003 | 0.035 |
| Ma — primary mRS outcome reference | 90 | M | 25% permanent / 6-month temporary duration | -0.046 | -0.088 to -0.002 | 0.019 |
| Ma — primary mRS outcome reference | 90 | M | 50% permanent / 6-month temporary duration (reference) | -0.068 | -0.110 to -0.024 | 0.002 |
| Ma — primary mRS outcome reference | 90 | M | 50% permanent / 3-month temporary duration | -0.064 | -0.106 to -0.021 | 0.003 |
| Ma — primary mRS outcome reference | 90 | M | 100% permanent persistence boundary | -0.112 | -0.154 to -0.068 | 0.000 |
| Ma — primary mRS outcome reference | 90 | F | 25% permanent / 3-month temporary duration | -0.030 | -0.084 to +0.031 | 0.151 |
| Ma — primary mRS outcome reference | 90 | F | 25% permanent / 6-month temporary duration | -0.036 | -0.090 to +0.025 | 0.114 |
| Ma — primary mRS outcome reference | 90 | F | 50% permanent / 6-month temporary duration (reference) | -0.064 | -0.118 to -0.003 | 0.021 |
| Ma — primary mRS outcome reference | 90 | F | 50% permanent / 3-month temporary duration | -0.060 | -0.114 to +0.001 | 0.026 |
| Ma — primary mRS outcome reference | 90 | F | 100% permanent persistence boundary | -0.120 | -0.174 to -0.059 | 0.000 |
| Catapano — treated, mixed severity | 80 | M | 25% permanent / 3-month temporary duration | +0.225 | +0.083 to +0.399 | 0.999 |
| Catapano — treated, mixed severity | 80 | M | 25% permanent / 6-month temporary duration | +0.218 | +0.077 to +0.393 | 0.999 |
| Catapano — treated, mixed severity | 80 | M | 50% permanent / 6-month temporary duration (reference) | +0.165 | +0.023 to +0.339 | 0.990 |
| Catapano — treated, mixed severity | 80 | M | 50% permanent / 3-month temporary duration | +0.169 | +0.027 to +0.343 | 0.992 |
| Catapano — treated, mixed severity | 80 | M | 100% permanent persistence boundary | +0.058 | -0.085 to +0.232 | 0.766 |
| Catapano — treated, mixed severity | 80 | F | 25% permanent / 3-month temporary duration | +0.358 | +0.162 to +0.599 | 1.000 |
| Catapano — treated, mixed severity | 80 | F | 25% permanent / 6-month temporary duration | +0.352 | +0.155 to +0.592 | 1.000 |
| Catapano — treated, mixed severity | 80 | F | 50% permanent / 6-month temporary duration (reference) | +0.285 | +0.089 to +0.526 | 0.999 |
| Catapano — treated, mixed severity | 80 | F | 50% permanent / 3-month temporary duration | +0.289 | +0.093 to +0.530 | 0.999 |
| Catapano — treated, mixed severity | 80 | F | 100% permanent persistence boundary | +0.152 | -0.045 to +0.394 | 0.931 |
| Catapano — treated, mixed severity | 85 | M | 25% permanent / 3-month temporary duration | +0.083 | +0.002 to +0.179 | 0.978 |
| Catapano — treated, mixed severity | 85 | M | 25% permanent / 6-month temporary duration | +0.077 | -0.004 to +0.173 | 0.969 |
| Catapano — treated, mixed severity | 85 | M | 50% permanent / 6-month temporary duration (reference) | +0.041 | -0.039 to +0.138 | 0.834 |
| Catapano — treated, mixed severity | 85 | M | 50% permanent / 3-month temporary duration | +0.045 | -0.035 to +0.142 | 0.858 |
| Catapano — treated, mixed severity | 85 | M | 100% permanent persistence boundary | -0.029 | -0.110 to +0.068 | 0.266 |
| Catapano — treated, mixed severity | 85 | F | 25% permanent / 3-month temporary duration | +0.152 | +0.042 to +0.289 | 0.996 |
| Catapano — treated, mixed severity | 85 | F | 25% permanent / 6-month temporary duration | +0.146 | +0.036 to +0.283 | 0.995 |
| Catapano — treated, mixed severity | 85 | F | 50% permanent / 6-month temporary duration (reference) | +0.101 | -0.009 to +0.238 | 0.961 |
| Catapano — treated, mixed severity | 85 | F | 50% permanent / 3-month temporary duration | +0.105 | -0.005 to +0.242 | 0.968 |
| Catapano — treated, mixed severity | 85 | F | 100% permanent persistence boundary | +0.011 | -0.099 to +0.149 | 0.572 |
| Catapano — treated, mixed severity | 90 | M | 25% permanent / 3-month temporary duration | +0.012 | -0.033 to +0.062 | 0.694 |
| Catapano — treated, mixed severity | 90 | M | 25% permanent / 6-month temporary duration | +0.007 | -0.039 to +0.056 | 0.606 |
| Catapano — treated, mixed severity | 90 | M | 50% permanent / 6-month temporary duration (reference) | -0.015 | -0.061 to +0.034 | 0.259 |
| Catapano — treated, mixed severity | 90 | M | 50% permanent / 3-month temporary duration | -0.012 | -0.057 to +0.038 | 0.312 |
| Catapano — treated, mixed severity | 90 | M | 100% permanent persistence boundary | -0.060 | -0.105 to -0.010 | 0.009 |
| Catapano — treated, mixed severity | 90 | F | 25% permanent / 3-month temporary duration | +0.038 | -0.021 to +0.108 | 0.892 |
| Catapano — treated, mixed severity | 90 | F | 25% permanent / 6-month temporary duration | +0.033 | -0.027 to +0.102 | 0.851 |
| Catapano — treated, mixed severity | 90 | F | 50% permanent / 6-month temporary duration (reference) | +0.005 | -0.055 to +0.074 | 0.555 |
| Catapano — treated, mixed severity | 90 | F | 50% permanent / 3-month temporary duration | +0.008 | -0.051 to +0.078 | 0.597 |
| Catapano — treated, mixed severity | 90 | F | 100% permanent persistence boundary | -0.052 | -0.111 to +0.018 | 0.068 |
| Goldberg — poor-grade | 80 | M | 25% permanent / 3-month temporary duration | +0.334 | +0.167 to +0.553 | 1.000 |
| Goldberg — poor-grade | 80 | M | 25% permanent / 6-month temporary duration | +0.328 | +0.161 to +0.547 | 1.000 |
| Goldberg — poor-grade | 80 | M | 50% permanent / 6-month temporary duration (reference) | +0.274 | +0.107 to +0.493 | 1.000 |
| Goldberg — poor-grade | 80 | M | 50% permanent / 3-month temporary duration | +0.278 | +0.111 to +0.498 | 1.000 |
| Goldberg — poor-grade | 80 | M | 100% permanent persistence boundary | +0.167 | -0.001 to +0.387 | 0.975 |
| Goldberg — poor-grade | 80 | F | 25% permanent / 3-month temporary duration | +0.515 | +0.283 to +0.814 | 1.000 |
| Goldberg — poor-grade | 80 | F | 25% permanent / 6-month temporary duration | +0.508 | +0.277 to +0.808 | 1.000 |
| Goldberg — poor-grade | 80 | F | 50% permanent / 6-month temporary duration (reference) | +0.441 | +0.210 to +0.741 | 1.000 |
| Goldberg — poor-grade | 80 | F | 50% permanent / 3-month temporary duration | +0.446 | +0.214 to +0.746 | 1.000 |
| Goldberg — poor-grade | 80 | F | 100% permanent persistence boundary | +0.308 | +0.076 to +0.609 | 0.996 |
| Goldberg — poor-grade | 85 | M | 25% permanent / 3-month temporary duration | +0.143 | +0.047 to +0.259 | 0.999 |
| Goldberg — poor-grade | 85 | M | 25% permanent / 6-month temporary duration | +0.137 | +0.041 to +0.253 | 0.998 |
| Goldberg — poor-grade | 85 | M | 50% permanent / 6-month temporary duration (reference) | +0.102 | +0.006 to +0.218 | 0.981 |
| Goldberg — poor-grade | 85 | M | 50% permanent / 3-month temporary duration | +0.106 | +0.010 to +0.222 | 0.984 |
| Goldberg — poor-grade | 85 | M | 100% permanent persistence boundary | +0.031 | -0.065 to +0.148 | 0.727 |
| Goldberg — poor-grade | 85 | F | 25% permanent / 3-month temporary duration | +0.237 | +0.106 to +0.402 | 1.000 |
| Goldberg — poor-grade | 85 | F | 25% permanent / 6-month temporary duration | +0.231 | +0.100 to +0.396 | 1.000 |
| Goldberg — poor-grade | 85 | F | 50% permanent / 6-month temporary duration (reference) | +0.186 | +0.055 to +0.352 | 0.998 |
| Goldberg — poor-grade | 85 | F | 50% permanent / 3-month temporary duration | +0.190 | +0.059 to +0.356 | 0.998 |
| Goldberg — poor-grade | 85 | F | 100% permanent persistence boundary | +0.097 | -0.035 to +0.262 | 0.918 |
| Goldberg — poor-grade | 90 | M | 25% permanent / 3-month temporary duration | +0.043 | -0.008 to +0.104 | 0.947 |
| Goldberg — poor-grade | 90 | M | 25% permanent / 6-month temporary duration | +0.038 | -0.014 to +0.099 | 0.922 |
| Goldberg — poor-grade | 90 | M | 50% permanent / 6-month temporary duration (reference) | +0.016 | -0.036 to +0.077 | 0.717 |
| Goldberg — poor-grade | 90 | M | 50% permanent / 3-month temporary duration | +0.020 | -0.032 to +0.080 | 0.762 |
| Goldberg — poor-grade | 90 | M | 100% permanent persistence boundary | -0.028 | -0.080 to +0.033 | 0.173 |
| Goldberg — poor-grade | 90 | F | 25% permanent / 3-month temporary duration | +0.082 | +0.014 to +0.168 | 0.991 |
| Goldberg — poor-grade | 90 | F | 25% permanent / 6-month temporary duration | +0.077 | +0.008 to +0.162 | 0.987 |
| Goldberg — poor-grade | 90 | F | 50% permanent / 6-month temporary duration (reference) | +0.049 | -0.020 to +0.134 | 0.905 |
| Goldberg — poor-grade | 90 | F | 50% permanent / 3-month temporary duration | +0.052 | -0.016 to +0.138 | 0.924 |
| Goldberg — poor-grade | 90 | F | 100% permanent persistence boundary | -0.008 | -0.076 to +0.078 | 0.426 |

1. The table displays 90 panel–setting rows. The reference rows are repeated for comparison and are the same 18 reference evaluations shown in Table 4; they are not additional evaluations. The nonreference portion comprises 72 executed evaluations.

2. The disposition-based family is not included because the registered structural sensitivity layers apply to the modified Rankin Scale-based panels only.

3. Probabilities are rounded to three decimals.

4. The reference configuration plus the three settings with 25% or 50% permanent morbidity constitute the restricted recovery comparison. The 100% permanent setting is a persistence/continuity boundary and is reported separately from that restricted set.

5. Recovery fractions and temporary durations are amendment-specified structural values, not evidence-derived clinical probabilities or recovery times.
