# Supplementary material for: Observation versus coiling for unruptured intracranial aneurysms in octogenarians: A decision-analytic modeling study
Source: PLoS One. 2026 Sep 1;21(9):e0357374. doi: 10.1371/journal.pone.0357374 (PMC13533429; doi:10.1371/journal.pone.0357374)
Supplement: Supplementary file 5 [file pone.0357374.s005.docx]

**S5 Table. residual post-treatment rupture-risk sensitivity. ΔQALY is coiling minus observation; positive values favor coiling. Values are medians with 95% uncertainty intervals and P(ΔQALY > 0) from 10,000 probabilistic iterations, with QALYs discounted at 3% annually.**

| **Outcome source** | **Age** | **Sex** | **Setting** | **Median ΔQALY** | **95% uncertainty interval** | **P(ΔQALY > 0)** |
| --- | --- | --- | --- | --- | --- | --- |
| Ma — primary mRS outcome reference | 80 | M | 0% of natural-history rupture risk | +0.022 | -0.110 to +0.195 | 0.617 |
| Ma — primary mRS outcome reference | 80 | M | 5% of natural-history rupture risk | +0.008 | -0.119 to +0.172 | 0.542 |
| Ma — primary mRS outcome reference | 80 | M | 10% of natural-history rupture risk (reference) | -0.006 | -0.129 to +0.148 | 0.459 |
| Ma — primary mRS outcome reference | 80 | M | 20% of natural-history rupture risk | -0.034 | -0.148 to +0.104 | 0.296 |
| Ma — primary mRS outcome reference | 80 | F | 0% of natural-history rupture risk | +0.097 | -0.088 to +0.335 | 0.841 |
| Ma — primary mRS outcome reference | 80 | F | 5% of natural-history rupture risk | +0.076 | -0.101 to +0.300 | 0.790 |
| Ma — primary mRS outcome reference | 80 | F | 10% of natural-history rupture risk (reference) | +0.055 | -0.115 to +0.267 | 0.731 |
| Ma — primary mRS outcome reference | 80 | F | 20% of natural-history rupture risk | +0.013 | -0.141 to +0.202 | 0.564 |
| Ma — primary mRS outcome reference | 85 | M | 0% of natural-history rupture risk | -0.042 | -0.119 to +0.051 | 0.175 |
| Ma — primary mRS outcome reference | 85 | M | 5% of natural-history rupture risk | -0.049 | -0.123 to +0.040 | 0.133 |
| Ma — primary mRS outcome reference | 85 | M | 10% of natural-history rupture risk (reference) | -0.055 | -0.128 to +0.029 | 0.093 |
| Ma — primary mRS outcome reference | 85 | M | 20% of natural-history rupture risk | -0.069 | -0.136 to +0.008 | 0.038 |
| Ma — primary mRS outcome reference | 85 | F | 0% of natural-history rupture risk | -0.010 | -0.116 to +0.120 | 0.434 |
| Ma — primary mRS outcome reference | 85 | F | 5% of natural-history rupture risk | -0.020 | -0.123 to +0.103 | 0.362 |
| Ma — primary mRS outcome reference | 85 | F | 10% of natural-history rupture risk (reference) | -0.031 | -0.130 to +0.086 | 0.290 |
| Ma — primary mRS outcome reference | 85 | F | 20% of natural-history rupture risk | -0.051 | -0.144 to +0.054 | 0.167 |
| Ma — primary mRS outcome reference | 90 | M | 0% of natural-history rupture risk | -0.062 | -0.106 to -0.015 | 0.005 |
| Ma — primary mRS outcome reference | 90 | M | 5% of natural-history rupture risk | -0.065 | -0.108 to -0.020 | 0.003 |
| Ma — primary mRS outcome reference | 90 | M | 10% of natural-history rupture risk (reference) | -0.068 | -0.110 to -0.024 | 0.002 |
| Ma — primary mRS outcome reference | 90 | M | 20% of natural-history rupture risk | -0.073 | -0.113 to -0.034 | 0.000 |
| Ma — primary mRS outcome reference | 90 | F | 0% of natural-history rupture risk | -0.055 | -0.113 to +0.011 | 0.051 |
| Ma — primary mRS outcome reference | 90 | F | 5% of natural-history rupture risk | -0.060 | -0.115 to +0.005 | 0.033 |
| Ma — primary mRS outcome reference | 90 | F | 10% of natural-history rupture risk (reference) | -0.064 | -0.118 to -0.003 | 0.021 |
| Ma — primary mRS outcome reference | 90 | F | 20% of natural-history rupture risk | -0.073 | -0.124 to -0.017 | 0.006 |
| Catapano — treated, mixed severity | 80 | M | 0% of natural-history rupture risk | +0.214 | +0.058 to +0.408 | 0.998 |
| Catapano — treated, mixed severity | 80 | M | 5% of natural-history rupture risk | +0.189 | +0.040 to +0.374 | 0.995 |
| Catapano — treated, mixed severity | 80 | M | 10% of natural-history rupture risk (reference) | +0.165 | +0.023 to +0.339 | 0.990 |
| Catapano — treated, mixed severity | 80 | M | 20% of natural-history rupture risk | +0.117 | -0.013 to +0.271 | 0.960 |
| Catapano — treated, mixed severity | 80 | F | 0% of natural-history rupture risk | +0.356 | +0.138 to +0.626 | 1.000 |
| Catapano — treated, mixed severity | 80 | F | 5% of natural-history rupture risk | +0.320 | +0.114 to +0.576 | 1.000 |
| Catapano — treated, mixed severity | 80 | F | 10% of natural-history rupture risk (reference) | +0.285 | +0.089 to +0.526 | 0.999 |
| Catapano — treated, mixed severity | 80 | F | 20% of natural-history rupture risk | +0.216 | +0.038 to +0.430 | 0.993 |
| Catapano — treated, mixed severity | 85 | M | 0% of natural-history rupture risk | +0.066 | -0.021 to +0.174 | 0.927 |
| Catapano — treated, mixed severity | 85 | M | 5% of natural-history rupture risk | +0.054 | -0.030 to +0.156 | 0.887 |
| Catapano — treated, mixed severity | 85 | M | 10% of natural-history rupture risk (reference) | +0.041 | -0.039 to +0.138 | 0.834 |
| Catapano — treated, mixed severity | 85 | M | 20% of natural-history rupture risk | +0.017 | -0.057 to +0.104 | 0.664 |
| Catapano — treated, mixed severity | 85 | F | 0% of natural-history rupture risk | +0.137 | +0.018 to +0.290 | 0.989 |
| Catapano — treated, mixed severity | 85 | F | 5% of natural-history rupture risk | +0.119 | +0.004 to +0.264 | 0.978 |
| Catapano — treated, mixed severity | 85 | F | 10% of natural-history rupture risk (reference) | +0.101 | -0.009 to +0.238 | 0.961 |
| Catapano — treated, mixed severity | 85 | F | 20% of natural-history rupture risk | +0.065 | -0.036 to +0.188 | 0.886 |
| Catapano — treated, mixed severity | 90 | M | 0% of natural-history rupture risk | -0.004 | -0.052 to +0.050 | 0.435 |
| Catapano — treated, mixed severity | 90 | M | 5% of natural-history rupture risk | -0.010 | -0.056 to +0.042 | 0.346 |
| Catapano — treated, mixed severity | 90 | M | 10% of natural-history rupture risk (reference) | -0.015 | -0.061 to +0.034 | 0.259 |
| Catapano — treated, mixed severity | 90 | M | 20% of natural-history rupture risk | -0.027 | -0.069 to +0.019 | 0.120 |
| Catapano — treated, mixed severity | 90 | F | 0% of natural-history rupture risk | +0.021 | -0.042 to +0.097 | 0.732 |
| Catapano — treated, mixed severity | 90 | F | 5% of natural-history rupture risk | +0.013 | -0.048 to +0.086 | 0.649 |
| Catapano — treated, mixed severity | 90 | F | 10% of natural-history rupture risk (reference) | +0.005 | -0.055 to +0.074 | 0.555 |
| Catapano — treated, mixed severity | 90 | F | 20% of natural-history rupture risk | -0.012 | -0.067 to +0.051 | 0.347 |
| Goldberg — poor-grade | 80 | M | 0% of natural-history rupture risk | +0.335 | +0.151 to +0.582 | 1.000 |
| Goldberg — poor-grade | 80 | M | 5% of natural-history rupture risk | +0.305 | +0.129 to +0.537 | 1.000 |
| Goldberg — poor-grade | 80 | M | 10% of natural-history rupture risk (reference) | +0.274 | +0.107 to +0.493 | 1.000 |
| Goldberg — poor-grade | 80 | M | 20% of natural-history rupture risk | +0.213 | +0.062 to +0.406 | 0.998 |
| Goldberg — poor-grade | 80 | F | 0% of natural-history rupture risk | +0.531 | +0.274 to +0.868 | 1.000 |
| Goldberg — poor-grade | 80 | F | 5% of natural-history rupture risk | +0.486 | +0.242 to +0.804 | 1.000 |
| Goldberg — poor-grade | 80 | F | 10% of natural-history rupture risk (reference) | +0.441 | +0.210 to +0.741 | 1.000 |
| Goldberg — poor-grade | 80 | F | 20% of natural-history rupture risk | +0.355 | +0.147 to +0.618 | 1.000 |
| Goldberg — poor-grade | 85 | M | 0% of natural-history rupture risk | +0.133 | +0.029 to +0.263 | 0.994 |
| Goldberg — poor-grade | 85 | M | 5% of natural-history rupture risk | +0.118 | +0.017 to +0.241 | 0.990 |
| Goldberg — poor-grade | 85 | M | 10% of natural-history rupture risk (reference) | +0.102 | +0.006 to +0.218 | 0.981 |
| Goldberg — poor-grade | 85 | M | 20% of natural-history rupture risk | +0.071 | -0.017 to +0.174 | 0.938 |
| Goldberg — poor-grade | 85 | F | 0% of natural-history rupture risk | +0.233 | +0.088 to +0.418 | 0.999 |
| Goldberg — poor-grade | 85 | F | 5% of natural-history rupture risk | +0.209 | +0.072 to +0.385 | 0.999 |
| Goldberg — poor-grade | 85 | F | 10% of natural-history rupture risk (reference) | +0.186 | +0.055 to +0.352 | 0.998 |
| Goldberg — poor-grade | 85 | F | 20% of natural-history rupture risk | +0.141 | +0.022 to +0.287 | 0.990 |
| Goldberg — poor-grade | 90 | M | 0% of natural-history rupture risk | +0.031 | -0.025 to +0.098 | 0.853 |
| Goldberg — poor-grade | 90 | M | 5% of natural-history rupture risk | +0.023 | -0.031 to +0.087 | 0.794 |
| Goldberg — poor-grade | 90 | M | 10% of natural-history rupture risk (reference) | +0.016 | -0.036 to +0.077 | 0.717 |
| Goldberg — poor-grade | 90 | M | 20% of natural-history rupture risk | +0.001 | -0.047 to +0.056 | 0.513 |
| Goldberg — poor-grade | 90 | F | 0% of natural-history rupture risk | +0.070 | -0.004 to +0.165 | 0.967 |
| Goldberg — poor-grade | 90 | F | 5% of natural-history rupture risk | +0.059 | -0.012 to +0.150 | 0.942 |
| Goldberg — poor-grade | 90 | F | 10% of natural-history rupture risk (reference) | +0.049 | -0.020 to +0.134 | 0.905 |
| Goldberg — poor-grade | 90 | F | 20% of natural-history rupture risk | +0.027 | -0.036 to +0.104 | 0.787 |

1. The table displays 72 panel–setting rows. The reference rows are repeated for comparison and are the same 18 reference evaluations shown in Table 4; they are not additional evaluations. The nonreference portion comprises 54 executed evaluations.

2. The disposition-based family is not included because the registered structural sensitivity layers apply to the modified Rankin Scale-based panels only.

3. Probabilities are rounded to three decimals.

4. The 10% reference is an uncalibrated structural assumption. The 0%, 5%, 10%, and 20% levels were specified in Protocol Amendment v1.5 before the extension results were generated; this analysis assesses sensitivity and does not empirically recalibrate the residual-risk parameter.
