# Supplementary material for: Observation versus coiling for unruptured intracranial aneurysms in octogenarians: A decision-analytic modeling study
Source: PLoS One. 2026 Sep 1;21(9):e0357374. doi: 10.1371/journal.pone.0357374 (PMC13533429; doi:10.1371/journal.pone.0357374)
Supplement: Supplementary file 6 [file pone.0357374.s006.docx]

**S6 Table. periprocedural coiling-morbidity sensitivity. ΔQALY is coiling minus observation; positive values favor coiling. Values are medians with 95% uncertainty intervals and P(ΔQALY > 0) from 10,000 probabilistic iterations, with QALYs discounted at 3% annually.**

| **Outcome source** | **Age** | **Sex** | **Setting** | **Median ΔQALY** | **95% uncertainty interval** | **P(ΔQALY > 0)** |
| --- | --- | --- | --- | --- | --- | --- |
| Ma — primary mRS outcome reference | 80 | M | 9.8% nonfatal morbidity (reference anchor) | -0.006 | -0.129 to +0.148 | 0.459 |
| Ma — primary mRS outcome reference | 80 | M | 5% nonfatal morbidity | +0.055 | -0.068 to +0.209 | 0.795 |
| Ma — primary mRS outcome reference | 80 | M | 3% nonfatal morbidity | +0.080 | -0.043 to +0.234 | 0.892 |
| Ma — primary mRS outcome reference | 80 | F | 9.8% nonfatal morbidity (reference anchor) | +0.055 | -0.115 to +0.267 | 0.731 |
| Ma — primary mRS outcome reference | 80 | F | 5% nonfatal morbidity | +0.129 | -0.042 to +0.340 | 0.930 |
| Ma — primary mRS outcome reference | 80 | F | 3% nonfatal morbidity | +0.159 | -0.011 to +0.371 | 0.968 |
| Ma — primary mRS outcome reference | 85 | M | 9.8% nonfatal morbidity (reference anchor) | -0.055 | -0.128 to +0.029 | 0.093 |
| Ma — primary mRS outcome reference | 85 | M | 5% nonfatal morbidity | -0.013 | -0.085 to +0.071 | 0.370 |
| Ma — primary mRS outcome reference | 85 | M | 3% nonfatal morbidity | +0.005 | -0.067 to +0.089 | 0.548 |
| Ma — primary mRS outcome reference | 85 | F | 9.8% nonfatal morbidity (reference anchor) | -0.031 | -0.130 to +0.086 | 0.290 |
| Ma — primary mRS outcome reference | 85 | F | 5% nonfatal morbidity | +0.021 | -0.078 to +0.138 | 0.655 |
| Ma — primary mRS outcome reference | 85 | F | 3% nonfatal morbidity | +0.043 | -0.057 to +0.160 | 0.788 |
| Ma — primary mRS outcome reference | 90 | M | 9.8% nonfatal morbidity (reference anchor) | -0.068 | -0.110 to -0.024 | 0.002 |
| Ma — primary mRS outcome reference | 90 | M | 5% nonfatal morbidity | -0.039 | -0.081 to +0.004 | 0.040 |
| Ma — primary mRS outcome reference | 90 | M | 3% nonfatal morbidity | -0.027 | -0.069 to +0.016 | 0.114 |
| Ma — primary mRS outcome reference | 90 | F | 9.8% nonfatal morbidity (reference anchor) | -0.064 | -0.118 to -0.003 | 0.021 |
| Ma — primary mRS outcome reference | 90 | F | 5% nonfatal morbidity | -0.029 | -0.083 to +0.032 | 0.160 |
| Ma — primary mRS outcome reference | 90 | F | 3% nonfatal morbidity | -0.015 | -0.069 to +0.046 | 0.303 |
| Catapano — treated, mixed severity | 80 | M | 9.8% nonfatal morbidity (reference anchor) | +0.165 | +0.023 to +0.339 | 0.990 |
| Catapano — treated, mixed severity | 80 | M | 5% nonfatal morbidity | +0.226 | +0.084 to +0.400 | 0.999 |
| Catapano — treated, mixed severity | 80 | M | 3% nonfatal morbidity | +0.251 | +0.110 to +0.425 | 1.000 |
| Catapano — treated, mixed severity | 80 | F | 9.8% nonfatal morbidity (reference anchor) | +0.285 | +0.089 to +0.526 | 0.999 |
| Catapano — treated, mixed severity | 80 | F | 5% nonfatal morbidity | +0.359 | +0.162 to +0.599 | 1.000 |
| Catapano — treated, mixed severity | 80 | F | 3% nonfatal morbidity | +0.390 | +0.193 to +0.630 | 1.000 |
| Catapano — treated, mixed severity | 85 | M | 9.8% nonfatal morbidity (reference anchor) | +0.041 | -0.039 to +0.138 | 0.834 |
| Catapano — treated, mixed severity | 85 | M | 5% nonfatal morbidity | +0.084 | +0.003 to +0.180 | 0.979 |
| Catapano — treated, mixed severity | 85 | M | 3% nonfatal morbidity | +0.101 | +0.021 to +0.198 | 0.993 |
| Catapano — treated, mixed severity | 85 | F | 9.8% nonfatal morbidity (reference anchor) | +0.101 | -0.009 to +0.238 | 0.961 |
| Catapano — treated, mixed severity | 85 | F | 5% nonfatal morbidity | +0.153 | +0.042 to +0.290 | 0.996 |
| Catapano — treated, mixed severity | 85 | F | 3% nonfatal morbidity | +0.174 | +0.064 to +0.311 | 0.999 |
| Catapano — treated, mixed severity | 90 | M | 9.8% nonfatal morbidity (reference anchor) | -0.015 | -0.061 to +0.034 | 0.259 |
| Catapano — treated, mixed severity | 90 | M | 5% nonfatal morbidity | +0.013 | -0.032 to +0.063 | 0.713 |
| Catapano — treated, mixed severity | 90 | M | 3% nonfatal morbidity | +0.025 | -0.020 to +0.075 | 0.861 |
| Catapano — treated, mixed severity | 90 | F | 9.8% nonfatal morbidity (reference anchor) | +0.005 | -0.055 to +0.074 | 0.555 |
| Catapano — treated, mixed severity | 90 | F | 5% nonfatal morbidity | +0.039 | -0.020 to +0.109 | 0.899 |
| Catapano — treated, mixed severity | 90 | F | 3% nonfatal morbidity | +0.054 | -0.005 to +0.123 | 0.960 |
| Goldberg — poor-grade | 80 | M | 9.8% nonfatal morbidity (reference anchor) | +0.274 | +0.107 to +0.493 | 1.000 |
| Goldberg — poor-grade | 80 | M | 5% nonfatal morbidity | +0.335 | +0.168 to +0.554 | 1.000 |
| Goldberg — poor-grade | 80 | M | 3% nonfatal morbidity | +0.360 | +0.193 to +0.579 | 1.000 |
| Goldberg — poor-grade | 80 | F | 9.8% nonfatal morbidity (reference anchor) | +0.441 | +0.210 to +0.741 | 1.000 |
| Goldberg — poor-grade | 80 | F | 5% nonfatal morbidity | +0.515 | +0.284 to +0.815 | 1.000 |
| Goldberg — poor-grade | 80 | F | 3% nonfatal morbidity | +0.546 | +0.315 to +0.845 | 1.000 |
| Goldberg — poor-grade | 85 | M | 9.8% nonfatal morbidity (reference anchor) | +0.102 | +0.006 to +0.218 | 0.981 |
| Goldberg — poor-grade | 85 | M | 5% nonfatal morbidity | +0.144 | +0.048 to +0.260 | 0.999 |
| Goldberg — poor-grade | 85 | M | 3% nonfatal morbidity | +0.162 | +0.066 to +0.278 | 1.000 |
| Goldberg — poor-grade | 85 | F | 9.8% nonfatal morbidity (reference anchor) | +0.186 | +0.055 to +0.352 | 0.998 |
| Goldberg — poor-grade | 85 | F | 5% nonfatal morbidity | +0.238 | +0.106 to +0.403 | 1.000 |
| Goldberg — poor-grade | 85 | F | 3% nonfatal morbidity | +0.259 | +0.128 to +0.424 | 1.000 |
| Goldberg — poor-grade | 90 | M | 9.8% nonfatal morbidity (reference anchor) | +0.016 | -0.036 to +0.077 | 0.717 |
| Goldberg — poor-grade | 90 | M | 5% nonfatal morbidity | +0.045 | -0.007 to +0.105 | 0.952 |
| Goldberg — poor-grade | 90 | M | 3% nonfatal morbidity | +0.057 | +0.005 to +0.117 | 0.985 |
| Goldberg — poor-grade | 90 | F | 9.8% nonfatal morbidity (reference anchor) | +0.049 | -0.020 to +0.134 | 0.905 |
| Goldberg — poor-grade | 90 | F | 5% nonfatal morbidity | +0.083 | +0.015 to +0.169 | 0.992 |
| Goldberg — poor-grade | 90 | F | 3% nonfatal morbidity | +0.098 | +0.030 to +0.183 | 0.998 |

1. The table displays 54 panel–setting rows. The reference rows are repeated for comparison and are the same 18 reference evaluations shown in Table 4; they are not additional evaluations. The nonreference portion comprises 36 executed evaluations.

2. The disposition-based family is not included because the registered structural sensitivity layers apply to the modified Rankin Scale-based panels only.

3. Probabilities are rounded to three decimals.

4. The 9.8% reference morbidity anchor derives from the submitted historical treatment-risk input. The 5% and 3% alternatives were specified in Protocol Amendment v1.5 before the extension results were generated; this remains a post hoc revision sensitivity rather than a replacement estimate of contemporary octogenarian coiling morbidity.

5. Periprocedural coiling mortality retained the submitted legacy probabilistic specification anchored at 2.4% and was not varied as a separate sensitivity parameter.
