# Supplementary material for: Observation versus coiling for unruptured intracranial aneurysms in octogenarians: A decision-analytic modeling study
Source: PLoS One. 2026 Sep 1;21(9):e0357374. doi: 10.1371/journal.pone.0357374 (PMC13533429; doi:10.1371/journal.pone.0357374)
Supplement: Supplementary file 7 [file pone.0357374.s010.docx]

**Manuscript ID:** ONE-D-26-30052 **Title:** Observation versus coiling for unruptured intracranial aneurysms in octogenarians: A decision-analytic modeling study

Dear Academic Editor and Reviewer,

We thank the Reviewer for identifying an important limitation in the original endpoint. That endpoint counted treatment morbidity, treatment death, rupture-related disability and rupture death as equivalent events despite substantial differences in severity, duration and reversibility. We have therefore revised the analysis itself rather than its interpretation.

The additions are a 24-panel severity-weighted quality-adjusted analysis; structural sensitivity analyses of recovery from treatment morbidity, residual post-treatment rupture risk and the historical coiling-morbidity anchor; component-specific outcome reporting; demotion of the threshold-zone classification to a secondary role; and a reorganized Discussion and Limitations. During revision, Protocol Amendment v1.5 was registered and frozen before any extension result was generated. Of 288 evaluations in that amended plan, 186 were executed; the remaining 102 were not, and this is reported as a deviation from the amended plan.

### Journal Requirements

**1. PLOS ONE style and file naming.** We reviewed the revised manuscript and Supporting Information for the requested PLOS ONE formatting and file-naming requirements. Supporting Information items are provided as separate files with names corresponding directly to their manuscript captions (S1 Table, S1 File, S2–S6 Tables, and S2 File), and clean and tracked versions of the revised manuscript are provided separately.

**2. Code sharing.** All author-generated analysis code underlying the reported findings, together with the corresponding reproducibility materials and per-evaluation outputs for the revision analyses, is openly available without restriction in the cited OSF repository (https://osf.io/vnzrt/). The journal-facing S1 File additionally contains the Python script and results table for the post hoc robustness analyses.

**3. Supporting life-table file.** We appreciate the journal’s concern regarding the previously submitted Korean_Life_Tables_qx_verified.xlsx file. The file was derived from publicly available aggregate national life-table statistics from the Korean Statistical Information Service (KOSIS), rather than from participant-level research data. Nevertheless, to avoid any ambiguity in the journal-facing Supporting Information, we replaced it with a minimized model-input workbook (S2 File). The minimized file contains model-cycle and panel identifiers with the qx values required to inspect the model inputs, but contains no single-year age column, sex labels, life-table survivor counts, life expectancy, or variables unused in the analysis. The exact age-indexed inputs used by the model remain preserved in the cited OSF reproducibility archive.

### Academic Editor

**1. Strengthen the Discussion with broader contextualization.** The Discussion is reorganized around interpretation: *Principal findings*, *Relation to prior literature and what this analysis adds*, *Structural sensitivity and parameter dependence*, and *Clinical interpretation*. It now frames the central result as conditionality rather than superiority. Of 30 endpoint-direction comparisons, 21 were concordant and nine discordant; all nine discordant comparisons shifted from observation toward coiling, but eight of the nine quality-adjusted intervals included zero.

**2. Consider DOI 10.1007/s11547-023-01620-x.** We reviewed Briganti et al. and cite it as reference 17. Because the study does not address contemporary octogenarian coiling outcomes, we use it specifically to illustrate device- and anatomy-specific planning in flow-diverter treatment. This supports our statement that the SAC/flow-diversion arm is a structural proxy rather than a calibrated estimate.

### Reviewer #1

**1. The endpoint gives equal weight to clinically unequal events.** We agree. The original endpoint is retained because it was the submitted primary analysis, but it is no longer the sole basis for clinical interpretation. The Abstract and Methods now state that it is an unweighted first-event composite in which the four components carry equal weight regardless of severity, duration or reversibility.

**2. Severity, duration and reversibility should be incorporated.** We added a 24-panel severity-weighted analysis discounted at 3% annually — 18 modified Rankin-based panels and six secondary disposition-based panels, reported by source rather than pooled. The revised Methods now specify the source-specific outcome distributions, the attained-age Korean EQ-5D baseline and Korean-tariff mRS utility mapping, the temporary-versus-permanent treatment-morbidity structure, QALY discounting, and the treatment of utility and procedural-risk inputs used to generate the analysis. ΔQALY ranged from −0.068 to +0.441; the 95% uncertainty interval excluded zero in eight of 18 modified Rankin-based panels and three of six disposition-based panels, with the remaining 13 spanning zero (Table 4). The revised Conclusions state that the observation-favoring pattern was not invariant to endpoint definition and that neither analysis establishes a universal preference.

**3. Recovery from treatment morbidity should be modeled.** We added 72 additional recovery evaluations. Within the reference and three restricted settings, median direction changed in three of 18 modified Rankin-based panels; the assumed permanent fraction mattered considerably more than shortening temporary morbidity from six months to three. A 100% permanent configuration is reported separately as a persistence boundary. These are an illustrative structural envelope, not estimated recovery probabilities — stated in Limitations L7, with panel-level results in S4 Table.

**4. Component-specific outcomes.** The four adverse first-event components are now reported separately for both strategies (S3 Table). In the Ma 80F panel, for instance, the coiling arm carried front-loaded treatment morbidity and mortality while the observation arm accumulated substantially more rupture death and rupture-related poor outcome; the unweighted endpoint counts each once. For the disposition family, rupture death and rupture-related nonfatal poor outcome cannot be separately identified from the source-defined occupancy trajectory and are reported as not identifiable rather than as zero, with total first ruptures given separately as a descriptive quantity.

**5. Residual rupture risk fixed at 10% without adequate calibration.** We agree, and we did not substitute an unsupported value. Amendment v1.5 specified 54 additional evaluations at 0%, 5% and 20% against the 10% reference. Median direction was unchanged in 16 of 18 panels; Ma 80M changed direction between 5% and 10% and Catapano 90F between 10% and 20%, with intervals including zero at every level in both. The manuscript now states that 10% remains an uncalibrated structural assumption and that the analysis quantifies dependence on it rather than establishing its value (Table 5, S5 Table, Limitations L4).

**6. Coiling morbidity rests on an older administrative dataset.** We agree. The 9.8% anchor derives from a U.S. National Inpatient Sample analysis of 2001–2008 admissions, and the Abstract now says so. We added 36 additional evaluations at 5% and 3% against the 9.8% reference. Median direction was unchanged in 14 of 18 panels; four changed direction, with intervals including zero at every level in all four. We do not present 5% or 3% as contemporary replacement estimates: the analysis quantifies dependence on the historical anchor rather than replacing it (Table 5, S6 Table, Limitations L8).

**7. Coiling mortality rests on the same dataset.** This concern remains unresolved. Targeted evidence review identified no contemporary, adequately powered estimate restricted to patients aged 80 years or older with an outcome definition compatible with immediate periprocedural mortality. The submitted 2.4% estimate was retained and was not varied in the new sensitivity analyses, and Limitations L8 states that the mortality anchor remains unresolved in the revised model.

**8. The post-rupture sources are clinically heterogeneous.** We agree and deliberately did not pool them. Ma serves as the primary modified Rankin reference; Catapano and Goldberg are distinct structural scenarios; Dasenbrock is a secondary disposition-based family. We also avoid any aggregate statement of the form “coiling was favored in X of 24 panels”, because such a count would imply an exchangeability that the analysis does not assume (Limitations L1).

**9. The evidence synthesis was not systematic and lacked risk-of-bias assessment.** We clarified this rather than overstating it. Registration and protocol amendments now states plainly that the manuscript is not a completed systematic review or meta-analysis, and that no meta-analysis, publication-bias assessment, formal risk-of-bias or certainty assessment, or dual-independent PRISMA-style screening was performed. The candidate worklist and verification record remain in the OSF archive.

**10. SAC/flow-diversion and vascular-access inputs are proxy or unanchored.** We agree. The SAC/flow-diversion arm is described as a structural proxy and the vascular-access analysis as an unanchored stress test; both are consolidated in Limitations L2 and neither determined the principal observation-versus-coiling comparison.

**11. Aneurysm- and patient-level determinants are omitted.** We agree, and this cannot be solved within an aggregate decision model; we did not add unsupported individualized adjustments. Clinical interpretation now states that the model does not represent aneurysm size, location, morphology, growth, previous subarachnoid hemorrhage, frailty, cognitive status or comorbidity, and therefore cannot support a recommendation for an individual patient. Limitations L9 states that it should be read as a parameterized decision framework rather than an aneurysm morphology-specific treatment calculator.

**12. Threshold zones risk converting continuous uncertainty into categories.** We agree. Continuous per-panel estimates and uncertainty intervals are now the primary results, with the zone classification retained only as a secondary summary. Table 3 is retitled *Secondary threshold-zone summary under the cumulative-incidence endpoint*; the former main-text table listing only coiling-favored zones is moved to S2 Table; and the horizontal lines in Fig 3 are identified as secondary interpretive aids. We did not introduce additional categorical equipoise bands because doing so would add further arbitrary cutoffs to the same continuous distributions and would reproduce rather than resolve the concern raised by the reviewer. The severity-weighted analysis therefore uses no threshold-zone classification or ±0.01 equipoise band and instead reports continuous ΔQALY estimates, 95% uncertainty intervals, and P(ΔQALY > 0). The separate rupture-risk and coiling-morbidity robustness analyses under the original endpoint remain sensitivity analyses of model assumptions rather than alternative categorical decision bands.

**13. The Abstract should state the endpoint and anchor limitations more clearly.** The Abstract now defines the original outcome as a first-event composite in which the four components carry equal weight regardless of severity, duration or reversibility; identifies the coiling inputs as originating from a U.S. National Inpatient Sample analysis of 2001–2008 and the residual rupture assumption as 10% of the natural-history rate; summarizes the severity-weighted analysis; and concludes that modeled preference is conditional on endpoint definition and structural assumptions.

**14. The Introduction and Methods were long and the Discussion repetitive.** The Introduction now closes by distinguishing the continuous primary outputs from the secondary threshold summary and introducing the extension. The Methods were streamlined by moving the event-ordering detail out of the Fig 1 legend and consolidating the software and registration material. The Discussion was reorganized around interpretation, with unresolved assumptions consolidated in the numbered Limitations.

### Closing

These revisions change what the model can properly be said to show. The revised manuscript no longer supports a simple conclusion that observation is generally preferred in octogenarians; it shows that the modeled direction depends on endpoint definition and on several structural assumptions, with the greatest instability in panels whose reference quality-adjusted differences are already close to zero. All eight modified Rankin-based panels whose reference intervals excluded zero retained their median direction across the three executed structural axes.

We have kept the unresolved limitations visible rather than absorbing them into the revision: the 10% residual rupture assumption remains uncalibrated, the coiling morbidity and mortality anchors remain historically dated, and the model does not contain the aneurysm- and patient-level information that an individual treatment decision requires.
