# Supplementary material for: Observation versus coiling for unruptured intracranial aneurysms in octogenarians: A decision-analytic modeling study
Source: PLoS One. 2026 Sep 1;21(9):e0357374. doi: 10.1371/journal.pone.0357374 (PMC13533429; doi:10.1371/journal.pone.0357374)
Supplement: Peer review history

# Peer review: Decision Letter 0

**DOI:** 10.1371/journal.pone.0357374.r001

**Authors:** Mario Tortora

15 Jul 2026

Dear Dr. YOO,

Thank you for submitting your manuscript to PLOS ONE. After careful consideration, we feel that it has merit but does not fully meet PLOS ONE’s publication criteria as it currently stands. Therefore, we invite you to submit a revised version of the manuscript that addresses the points raised during the review process.

Please submit your revised manuscript by Sep 13 2026 11:59PM. If you will need more time than this to complete your revisions, please reply to this message or contact the journal office at plosone@plos.org. When you're ready to submit your revision, log on to https://www.editorialmanager.com/pone/ and select the 'Submissions Needing Revision' folder to locate your manuscript file.

- A letter that responds to each point raised by the academic editor and reviewer(s). You should upload this letter as a separate file labeled 'Response to Reviewers'.
- A marked-up copy of your manuscript that highlights changes made to the original version. You should upload this as a separate file labeled 'Revised Manuscript with Track Changes'.
- An unmarked version of your revised paper without tracked changes. You should upload this as a separate file labeled 'Manuscript'.

If you would like to make changes to your financial disclosure, please include your updated statement in your cover letter. Guidelines for resubmitting your figure files are available below the reviewer comments at the end of this letter.

If applicable, we recommend that you deposit your laboratory protocols in protocols.io to enhance the reproducibility of your results. Protocols.io assigns your protocol its own identifier (DOI) so that it can be cited independently in the future. For instructions see: https://journals.plos.org/plosone/s/submission-guidelines#loc-laboratory-protocols. Additionally, PLOS ONE offers an option for publishing peer-reviewed Lab Protocol articles, which describe protocols hosted on protocols.io. Read more information on sharing protocols at https://plos.org/protocols?utm_medium=editorial-email&utm_source=authorletters&utm_campaign=protocols.

As the corresponding author, your ORCID iD is verified in the submission system and will appear in the published article. PLOS supports the use of ORCID, and we encourage all coauthors to register for an ORCID iD and use it as well. Please encourage your coauthors to verify their ORCID iD within the submission system before final acceptance, as unverified ORCID iDs will not appear in the published article. *Only*  the individual author can complete the verification step; PLOS staff *cannot*  verify ORCID iDs on behalf of authors.

We look forward to receiving your revised manuscript.

Kind regards,

Mario Tortora, Pd.D.s, M.D.

Academic Editor

PLOS One

**Journal Requirements:**

When submitting your revision, we need you to address these additional requirements.

1. Please ensure that your manuscript meets PLOS ONE's style requirements, including those for file naming. The PLOS ONE style templates can be found at

https://journals.plos.org/plosone/s/file?id=wjVg/PLOSOne_formatting_sample_main_body.pdf and

https://journals.plos.org/plosone/s/file?id=ba62/PLOSOne_formatting_sample_title_authors_affiliations.pdf

2. Please note that PLOS One has specific guidelines on code sharing for submissions in which author-generated code underpins the findings in the manuscript. In these cases, we expect all author-generated code to be made available without restrictions upon publication of the work. Please review our guidelines at https://journals.plos.org/plosone/s/materials-and-software-sharing#loc-sharing-code and ensure that your code is shared in a way that follows best practice and facilitates reproducibility and reuse.

3. We note that there is identifying data in the Supporting Information file < Korean_Life_Tables_qx_verified.xlsx>. Due to the inclusion of these potentially identifying data, we have removed this file from your file inventory. Prior to sharing human research participant data, authors should consult with an ethics committee to ensure data are shared in accordance with participant consent and all applicable local laws.

Data sharing should never compromise participant privacy. It is therefore not appropriate to publicly share personally identifiable data on human research participants. The following are examples of data that should not be shared:

-Name, initials, physical address

-Ages more specific than whole numbers

-Internet protocol (IP) address

-Specific dates (birth dates, death dates, examination dates, etc.)

-Contact information such as phone number or email address

-Location data

-ID numbers that seem specific (long numbers, include initials, titled “Hospital ID”) rather than random (small numbers in numerical order)

Data that are not directly identifying may also be inappropriate to share, as in combination they can become identifying. For example, data collected from a small group of participants, vulnerable populations, or private groups should not be shared if they involve indirect identifiers (such as sex, ethnicity, location, etc.) that may risk the identification of study participants.

Additional guidance on preparing raw data for publication can be found in our Data Policy (https://journals.plos.org/plosone/s/data-availability#loc-human-research-participant-data-and-other-sensitive-data) and in the following article: http://www.bmj.com/content/340/bmj.c181.long.

Please remove or anonymize all personal information ( specific age data in each sheet of column a), ensure that the data shared are in accordance with participant consent, and re-upload a fully anonymized data set. Please note that spreadsheet columns with personal information must be removed and not hidden as all hidden columns will appear in the published file.

If the reviewer comments include a recommendation to cite specific previously published works, please review and evaluate these publications to determine whether they are relevant and should be cited. There is no requirement to cite these works unless the editor has indicated otherwise.

**Additional Editor Comments:**

Dear Authors,

After careful consideration of the reviewer' comments, we believe that the manuscript has the potential to make a valuable contribution to the field.

However, before it can be considered for publication, we encourage you to carefully address all the points raised by the reviewer.

In particular, we recommend strengthening the Discussion section. While the current discussion adequately summarizes your findings, it would benefit from a broader contextualization within the existing literature, highlighting the clinical implications, methodological aspects, and the relevance of your results in comparison with previously published studies.

We also encourage you to expand the discussion by considering pertinent literature, including recent articles published in the field, such as 10.1007/s11547-023-01620-x, where appropriate to your work. Integrating relevant contemporary evidence will help place your findings in a wider scientific context and improve the overall impact of the manuscript.

We look forward to receiving a thoroughly revised version together with a detailed point-by-point response addressing each reviewer comment.

Sincerely,

The Academic Editor

Reviewers' comments:

Reviewer's Responses to Questions

**Comments to the Author**

1. Is the manuscript technically sound, and do the data support the conclusions?

Reviewer #1: Yes

**********

2. Has the statistical analysis been performed appropriately and rigorously?

Reviewer #1: Yes

**********

3. Have the authors made all data underlying the findings in their manuscript fully available?

The PLOS Data policy

Reviewer #1: Yes

**********

4. Is the manuscript presented in an intelligible fashion and written in standard English?

Reviewer #1: Yes

**********

Reviewer #1: This manuscript addresses an important and underexplored clinical dilemma: whether preventive coiling provides a meaningful advantage over observation for unruptured intracranial aneurysms in patients aged 80 years or older. The competing-risk Markov framework is appropriate in principle, because procedural harm, aneurysm rupture, post-rupture disability, and non-aneurysm mortality interact over a limited remaining lifespan. The analysis is unusually transparent regarding protocol amendments, source limitations, model assumptions, and the distinction between model-derived preferences and individualized treatment recommendations. The finding that observation was favored in most modeled panels is clinically interesting, but it should be interpreted as highly dependent on the selected endpoint and parameter assumptions rather than as robust evidence for conservative management.

The principal strengths are the explicit incorporation of age- and sex-specific competing mortality, the use of 10,000 probabilistic sensitivity-analysis iterations across multiple outcome scenarios, and the public availability of model code, parameter tables, and supporting outputs. Independent deterministic validation in Python and R strengthens confidence in the computational implementation. The decision to present heterogeneous post-rupture outcome estimates as separate scenarios rather than pooling nonexchangeable populations is methodologically defensible and appropriately exposes structural uncertainty. The authors also acknowledge that the apparent sex differences largely arise within demographic panels governed by differing life expectancy and competing mortality, and they consistently caution that the results are model outputs rather than direct clinical evidence.

The major concern is the clinical validity of the primary endpoint. An unweighted first-event cumulative incidence treats nonfatal treatment morbidity, treatment death, rupture-related disability, and rupture death as equivalent events, although their severity, duration, reversibility, and consequences differ substantially. Moreover, treatment morbidity appears to be retained permanently until other-cause death, without modeling recovery or changing disability over time. This structure can strongly disadvantage intervention because a front-loaded, potentially transient procedural morbidity is counted identically to permanent severe disability or death. A decision-analysis manuscript should therefore include a severity-weighted analysis, preferably using utilities or quality-adjusted life-years, or at minimum report each event component separately and examine plausible recovery trajectories. The current endpoint may be useful descriptively, but it is insufficient as the principal basis for inferring comparative clinical preference.

A second major concern is the fragility of the parameterization. Coiling morbidity and mortality are anchored to an older administrative dataset, the residual post-treatment rupture risk is fixed at 10% of the natural-history rate without adequate empirical calibration, and the post-rupture scenarios differ markedly in clinical grade, treatment status, outcome definition, and follow-up. The targeted evidence synthesis was not a systematic review and included no formal risk-of-bias or certainty assessment, while the SAC/flow-diversion and vascular-access inputs are explicitly proxy or unanchored constructs. The model also omits aneurysm size, location, morphology, growth, previous subarachnoid hemorrhage, frailty, cognitive status, and comorbidity, which are central determinants of both rupture and treatment risk. The arbitrary ±0.01 equipoise band and hierarchical threshold zones further risk converting continuous uncertainty into apparently definitive categories; continuous estimates and uncertainty intervals should remain primary, with alternative threshold analyses provided. The post hoc findings showing that coiling becomes favored in most panels when rupture risk rises or morbidity falls confirm that the central conclusion is highly assumption-sensitive.

The Introduction and Methods are detailed but overly long, and the Discussion repeats several methodological caveats that could be consolidated. The Abstract should more prominently state that the result is driven by an unweighted composite endpoint and historical treatment-risk estimates. Ethical handling is appropriate because only published aggregate data were used, and the open-data statement is a notable strength. Nevertheless, the manuscript requires substantial revision before its conclusions can be considered clinically interpretable. The authors should add severity-weighted or utility-based analyses, model temporary versus permanent morbidity, justify or recalibrate residual rupture assumptions, reduce reliance on categorical threshold zones, and present clearer component-specific outcomes. My recommendation is **major revision**.

**********

what does this mean?). If published, this will include your full peer review and any attached files.

If you choose “no”, your identity will remain anonymous but your review may still be made public.

**Do you want your identity to be public for this peer review?**  For information about this choice, including consent withdrawal, please see our Privacy Policy

Reviewer #1: No

**********

[NOTE: If reviewer comments were submitted as an attachment file, they will be attached to this email and accessible via the submission site. Please log into your account, locate the manuscript record, and check for the action link "View Attachments". If this link does not appear, there are no attachment files.]

To ensure your figures meet our technical requirements, please review our figure guidelines: https://journals.plos.org/plosone/s/figures

You may also use PLOS’s free figure tool, NAAS, to help you prepare publication quality figures: https://journals.plos.org/plosone/s/figures#loc-tools-for-figure-preparation.

NAAS will assess whether your figures meet our technical requirements by comparing each figure against our figure specifications.

# Author comment: Author response to Decision Letter 1

**DOI:** 10.1371/journal.pone.0357374.r002

15 Aug 2026

We thank the Academic Editor and Reviewer for their detailed and constructive comments. We have addressed each point in the revised manuscript and provide a full point-by-point response in the uploaded file Response_to_Reviewers.docx.

The principal revision is a 24-panel severity-weighted QALY analysis added in response to the concern that the original first-event endpoint gave equal weight to clinically unequal outcomes. We also added recovery-trajectory, residual post-treatment rupture-risk, and coiling-morbidity sensitivity analyses; component-specific outcome reporting; demoted threshold-zone classifications to a secondary role; expanded the Discussion and Limitations; and responded to the journal’s additional requirements regarding file formatting, code sharing, and the supporting life-table file, which we replaced with a minimized model-input workbook. Protocol Amendment v1.5 was registered and frozen before any extension results were generated, and deviations from the amended analysis plan are reported transparently.

Please see the attached Response_to_Reviewers.docx for our complete responses to all Editor, Reviewer, and Journal Requirement comments.

Attachment Submitted filename: Response_to_Reviewers.docx

# Editor report: Decision Letter 1

**DOI:** 10.1371/journal.pone.0357374.r003

**Authors:** Mario Tortora

17 Aug 2026

Observation versus coiling for unruptured intracranial aneurysms in octogenarians: A decision-analytic modeling study

PONE-D-26-30052R1

Dear Dr. HYUN DONG YOO,

We’re pleased to inform you that your manuscript has been judged scientifically suitable for publication and will be formally accepted for publication once it meets all outstanding technical requirements.

Within one week, you’ll receive an e-mail detailing the required amendments. When these have been addressed, you’ll receive a formal acceptance letter and your manuscript will be scheduled for publication.

An invoice will be generated when your article is formally accepted. Please note, if your institution has a publishing partnership with PLOS and your article meets the relevant criteria, all or part of your publication costs will be covered. Please make sure your user information is up-to-date by logging into Editorial Manager at Editorial Manager® and clicking the ‘Update My Information' link at the top of the page. For questions related to billing, please contact billing support.

If your institution or institutions have a press office, please notify them about your upcoming paper to help maximize its impact. If they’ll be preparing press materials, please inform our press team as soon as possible -- no later than 48 hours after receiving the formal acceptance. Your manuscript will remain under strict press embargo until 2 pm Eastern Time on the date of publication. For more information, please contact onepress@plos.org.

Kind regards,

Mario Tortora, PhD, M.D.

Academic Editor

PLOS One

Additional Editor Comments (optional):

The authors have adequately addressed all of the reviewers’ comments and concerns. The revised manuscript has been carefully evaluated, and I am satisfied that all requested revisions have been appropriately addressed. Therefore, I am pleased to recommend acceptance of the manuscript for publication in its present form.

Reviewers' comments:

# Editor report: Acceptance letter

**DOI:** 10.1371/journal.pone.0357374.r004

**Authors:** Mario Tortora

PONE-D-26-30052R1

PLOS One

Dear Dr. YOO,

I'm pleased to inform you that your manuscript has been deemed suitable for publication in PLOS One. Congratulations! Your manuscript is now being handed over to our production team.

At this stage, our production department will prepare your paper for publication. This includes ensuring the following:

* All references, tables, and figures are properly cited

* All relevant supporting information is included in the manuscript submission,

* There are no issues that prevent the paper from being properly typeset

You will receive further instructions from the production team, including instructions on how to review your proof when it is ready. Please keep in mind that we are working through a large volume of accepted articles, so please give us a few days to review your paper and let you know the next and final steps.

Lastly, if your institution or institutions have a press office, please let them know about your upcoming paper now to help maximize its impact. If they'll be preparing press materials, please inform our press team within the next 48 hours. Your manuscript will remain under strict press embargo until 2 pm Eastern Time on the date of publication. For more information, please contact onepress@plos.org.

You will receive an invoice from PLOS for your publication fee after your manuscript has reached the completed accept phase. If you receive an email requesting payment before acceptance or for any other service, this may be a phishing scheme. Learn how to identify phishing emails and protect your accounts at https://explore.plos.org/phishing.

If we can help with anything else, please email us at customercare@plos.org.

Thank you for submitting your work to PLOS One and supporting open access.

Kind regards,

PLOS ONE Editorial Office Staff

on behalf of

Dr. Mario Tortora

Academic Editor

PLOS One
